# On quivers, spectral networks and black holes

**DOI:** 10.1007/s11005-026-02136-x

**Published:** 2026-09-18

**Authors:** Paolo Arnaudo, Alba Grassi, Qianyu Hao

**Affiliations:** 1 Mathematical Sciences and STAG Research Centre, University of Southampton, Highfield, Southampton, SO17 1BJ UK; 2 Section de Mathématiques, Université de Genève, Geneva 4, 1211 Switzerland; 3 Theoretical Physics Department, CERN, Geneva 23, 1211 Switzerland

## Abstract

It was recently found that connection coefficients of the Heun equation can be derived in a closed form using crossing symmetry in two-dimensional Liouville theory via the Nekrasov–Shatashvili functions. In this work, we systematize this approach to second-order linear ODEs of Fuchsian type, which arise in the description of $\mathcal {N}=2$, four-dimensional quiver gauge theories. After presenting the general procedure, we focus on the specific case of Fuchsian equations with five regular singularities and present some applications to black-hole perturbation theory. First, we consider a massive scalar perturbation of the Schwarzschild black hole in AdS_7_. Next, we analyze vector-type perturbations of the Reissner–Nordström-AdS_5_ black hole. We also discuss the implications of our results in the context of the AdS/CFT correspondence and present explicit results in the large spin limit, where we make connection with the light-cone bootstrap. Furthermore, using the spectral network technology, we identify the region of the moduli space in Seiberg–Witten theory that is relevant for the study of black-hole quasinormal modes. Our results suggest that, in some cases, this region corresponds to the strong-coupling regime, highlighting the potential applicability of the conformal GMN TBA framework to address scenarios where the gravitational dictionary implies that the instanton counting parameters are not parametrically small.

## Introduction

In recent years, many connections have been uncovered between quantum spectral problems on the one hand and supersymmetric gauge theories or topological string theories on the other, see [1] for a review. An important result relevant to this work was presented in [2], where the authors found that the partition functions of four-dimensional, $\mathcal {N}=2$ gauge theories, evaluated in the Nekrasov–Shatashvili (NS) phase of the Ω background, serve as fundamental building blocks for constructing solutions to a class of differential equations known as four-dimensional quantum Seiberg–Witten (SW) curves. Initially, the works [2–6] primarily focused on the quantization conditions for the energy spectrum, while the study of eigenfunctions was performed in more details later, see for instance [7–15]. In [16, 17], a method was developed to obtain a closed-form expression for the Fredholm determinants of such operators by taking a special limit of the TS/ST correspondence [18]. More recently in [19], it was found that these determinants, and the associated connection coefficients, can be derived in an alternative way using crossing symmetry in two-dimensional Liouville theory,^1^ through the AGT correspondence [21]. In this context, the NS partition functions compute the semiclassical conformal blocks. In [19], the explicit example of Heun equation was worked out in detail. In this work, we build on this approach and extend it to second-order linear ODEs of Fuchsian type.^2^ After presenting the general procedure, we focus on the detailed analysis of a Fuchsian equation with five regular singularities.

One motivation for studying these problems arises from their connection to black-hole perturbation theory, which, as discussed in [22], can be systematically explored using the Nekrasov–Shatashvili functions. This framework has been effectively extended to a variety of gravitational backgrounds and has applications that go well beyond the computation of quasinormal modes (QNMs); see, for example, [22–39]. Several applications have also been discussed in the context of the AdS/CFT correspondence, as we discuss in Sect. 4. In the cases studied so far, the relevant differential equation has at most four (regular) singularities. However, especially in the context of higher-dimensional black holes, the underling differential equation can have an arbitrarily high number of singular points. This makes it interesting to perform a detailed analysis of such scenarios as well.

This paper is structured as follows: In Sect. 2, we present the detailed procedure for computing the connection coefficients of a second-order linear ODE of Fuchsian type, which corresponds to a four-dimensional $\mathcal {N}=2$ quiver theory. In general, if the equation has more than four singularities, then the relevant $\mathcal {N}=2$ theory can have multiple representations as a weakly coupled generalized quiver [40]. Here, we will mostly deal with linear quiver gauge theories.

In Sect. 3, we apply this analysis to the specific example of a Fuchsian equation with five regular singularities. A new feature for cases with more than four singularities is the appearance of non-comb-like diagrams, an example of which is presented in Sect. 3.3. In all cases, we formulate the problem in terms of Frobenius solutions and by using the full NS functions (including classical and one-loop). This enables us to express the ratio of connection coefficients as trigonometric functions with argument $F^\textrm{NS}$. We cross-check our results numerically. In Sect. 4, we explore some applications. Specifically, in Sect. 4.1, we examine a massive scalar perturbation of a Schwarzschild-AdS_7_ black hole, while in Sect. 4.2 we study gravitational and electromagnetic (vector-type) perturbation a Reissner–Nordström (RN) black hole in AdS_5_. We present a detailed analysis of the expansion at large $M/\ell ^{(d-2)/2}$ where we make contact with the light-cone bootstrap.

In Sect. 5, we initiate the study of spectral networks (SNs) within the framework of black-hole perturbation theory. Our findings suggest that some QNM spectral problems reside in the strong coupling region of the Seiberg–Witten moduli space. This observation opens the way for exploring alternative techniques, such as the conformal Gaiotto–Moore–Neitzke (GMN) TBA formalism [41, 42], to address scenarios like the hydrodynamic limit, where the gravitational dictionary indicates that the instanton counting parameters in the NS functions are not parametrically small. Building on these results, it would be important to gain a deeper understanding of the implications of wall-crossing phenomena on the gravity side, as well as to further investigate black-hole phase transitions on the spectral networks side. We hope to report on this in the future.

## The general strategy

### Liouville CFT

Among the 2d conformal field theories (CFT), an important model is the Liouville theory. In this section, we review the background of the 2d Liouville CFT. The vertex algebra of the Liouville CFT is the Virasoro algebra. The central charge *c* of the Virasoro algebra can be expressed in terms of the background charge *Q* or the coupling constant *b* as $c=1+6Q^2$, where Q=b+1/b. Liouville theory also depends on a Riemann surface *C*.

The spectrum of the theory is diagonal and continuous. The conformal dimensions of the primary fields are usually denoted by Δ, and they are parametrized by the momenta α as

2.1 $$\begin{aligned} \Delta =\frac{Q^2}{4}-\alpha ^2.\end{aligned}$$

Liouville CFT allows the existence of reducible representations of the Virasoro algebra. The invariant submodules are generated by null states, which have the property of being annihilated by all the positive generators of the local conformal transformations $L_n$, n>0. At level n=2, a reducible representation is generated by the degenerate field $\Phi _{2,1}$ with momentum $\alpha _{2,1}=-b-\frac{1}{2b}$ and conformal weight $\Delta _{2,1}=-\frac{1}{2}-\frac{3}{4}b^2$. The null state of $\Phi _{2,1}$ is its descendant

2.2 $$\begin{aligned} \left( b^{-2}L_{-1}^2+L_{-2}\right) |\Phi _{2,1}\rangle . \end{aligned}$$

Since null states decouple from correlation functions, the following equation is satisfied with all possible insertions of primary fields $V_{\alpha _i}$, i=0,⋯,n-1,

2.3 $$\begin{aligned} \left\langle \prod _{i=0}^{n-1}V_{\alpha _i}(z_i)\left( b^{-2}L_{-1}^2+L_{-2}\right) \Phi _{2,1}(z)\right\rangle =0, \end{aligned}$$

where $z_i\in C$. Using the local Ward identities, it is possible to write (2.3) as a partial differential equation known as BPZ equation [43]:

2.4 $$\begin{aligned} \left( \frac{1}{b^{2}}\frac{\partial ^{2}}{\partial z^{2}}+\sum _{i=0}^{n-1}\left( \frac{1}{z-z_{i}}\frac{\partial }{\partial z_{i}}+\frac{\Delta _{i}}{(z-z_{i})^{2}}\right) \right) \left\langle \prod _{i=0}^{n-1}V_{\alpha _{i}}(z_{i})\Phi _{2,1}(z)\right\rangle =0. \end{aligned}$$

We can assume without loss of generality that three of the insertion points, say $z_0, z_1, z_{n-1}$, are located at z=0,1,∞, and therefore represent the correlator as

2.5 $$\begin{aligned} \left\langle \Delta _{\infty }|V_{\alpha _1}(1)\prod _{i=2}^{n-2}V_{\alpha _i}(z_i)\Phi _{2,1}(z)|\Delta _0\right\rangle .\end{aligned}$$

The correlator (2.5) is calculated using the operator product expansion (OPE). When we perform the calculation such that there is an OPE between the degenerate field $\Phi _{2,1}(z)$ and one of the primary fields $V_{\alpha _i}(z_i)$,

2.6 $$\begin{aligned} \Phi _{2,1}(z)V_{\alpha _i}(z_i)=\sum _{\pm }C_{\alpha _{2,1}\alpha _i}^{\alpha _i\pm \frac{b}{2}}(z-z_i)^{\frac{bQ}{2}\mp \alpha _i}V_{\alpha _i\mp \frac{b}{2}}(z_i)+\mathcal {O}\left( (z-z_i)^{\frac{bQ}{2}\mp \alpha _i+1}\right) , \end{aligned}$$

inside the correlator, we obtain the *conformal block expansion* of a basis of local solutions of the BPZ equation for $z\sim z_i$. Here

2.7 $$\begin{aligned} \alpha _{i\theta }=\alpha _i- \theta \frac{b}{2}, \quad \theta =\pm 1~, \end{aligned}$$

2.8 $$\begin{aligned}&C_{\alpha _1\alpha _2\alpha _3} =\frac{\Upsilon '_b(0)\Upsilon _b(Q+2\alpha _1)\Upsilon _b(Q+2\alpha _2)\Upsilon _b(Q+2\alpha _3) \Upsilon _b^{-1}\big (\frac{Q}{2}+\alpha _1+\alpha _2+\alpha _3\big )}{\Upsilon _b\big (\frac{Q}{2}+\alpha _1+\alpha _2-\alpha _3\big )\Upsilon _b\big (\frac{Q}{2}+\alpha _1-\alpha _2+\alpha _3\big )\Upsilon _b\big (\frac{Q}{2}-\alpha _1+\alpha _2+\alpha _3\big )}, \end{aligned}$$

and

2.9 $$\begin{aligned} C^{\alpha _1}_{\alpha _2\alpha _3}=G_{\alpha _1}^{-1}C_{\alpha _1\alpha _2\alpha _3}, \end{aligned}$$

where

2.10 $$\begin{aligned} G_\alpha =\frac{\Upsilon _b(2\alpha +Q)}{\Upsilon _b(2\alpha )}. \end{aligned}$$

Equation (2.8) is known as the DOZZ formula [44, 45]; we refer to [46] for a review and a definition of the $\Upsilon _b$ function.

If we want to study the local solutions of (2.4) around z∼0, we use an OPE expansion of the form

2.11 $$\begin{aligned} \begin{aligned}&\left\langle \Delta _{\infty }\left| V_{\alpha _1}(1)\prod _{i=2}^{n-2}V_{\alpha _i}(z_i)\Phi _{2,1}(z)\right| \Delta _0\right\rangle \\&\quad =\sum _{\theta =\pm }\int \textrm{d}\beta _1\dots \textrm{d}\beta _{n-3}\,C_{\alpha _{2,1}\alpha _0}^{\alpha _{0\theta }}C_{\alpha _{n-2}\alpha _{0\theta }}^{\beta _1}\prod _{i=1}^{n-4}C^{\beta _{i+1}}_{\alpha _{n-i-2}\,\beta _i}C_{\alpha _{\infty }\alpha _1\beta _{n-3}}\\&\quad \quad \quad \times \bigg |\mathfrak {F}\bigg ( \begin{matrix} \alpha _{1} \\ \alpha _{\infty } \end{matrix} \, \beta _{n-3} \, \begin{matrix} \alpha _{2} \\ \, \end{matrix} \,\dots \, \beta _1\,\begin{matrix} \alpha _{n-2} \\ \, \end{matrix} \, \alpha _{0\theta } \, \begin{matrix} \alpha _{2,1} \\ \alpha _0\end{matrix};\frac{z_2}{z_1},\frac{z_3}{z_2}\cdots ,\frac{z_{n-2}}{z_{n-3}},\frac{z}{z_{n-2}}\bigg )\bigg |^2, \end{aligned} \end{aligned}$$

where $$\mathfrak {F}\bigg ( \begin{matrix} \alpha _{1} \\ \alpha _{\infty } \end{matrix} \, \beta _{n-3} \, \begin{matrix} \alpha _{2} \\ \, \end{matrix} \,\dots \, \beta _1\,\begin{matrix} \alpha _{n-2} \\ \, \end{matrix} \, \alpha _{0\theta } \, \begin{matrix} \alpha _{2,1} \\ \alpha _0\end{matrix};\frac{z_2}{z_1},\frac{z_3}{z_2}\cdots ,\frac{z_{n-2}}{z_{n-3}},\frac{z}{z_{n-2}}\bigg ) $$ is the (n+1)-point conformal block, and we assumed $|z_{n-2}|<|z_{n-3}|<\dots<|z_2|<1=z_1$. Diagrammatically, we represent this conformal block as a “comb” diagram2.12which gives an order for performing OPEs. To be more concrete, each trivalent component2.13represents an OPE

2.14 $$\begin{aligned} V_{\beta _l}(z)V_{\alpha _k}(z_k)= &   \int \textrm{d}{\beta _{l+1}}C_{\beta _l\alpha _k}^{\beta _{l+1}}(z-z_k)^{\Delta _{\beta _{l+1}}-\Delta _{\beta _l}-\Delta _{\alpha _k}}V_{\beta _{l+1}}(z_k)\nonumber \\  &   +\mathcal {O}\left( (z-z_k)^{\Delta _{\beta _{l+1}}-\Delta _{\beta _l}-\Delta _{\alpha _k}+1}\right) . \end{aligned}$$

An important scaling limit of the Liouville CFT is the *semiclassical limit* given by

2.15 $$\begin{aligned} b\rightarrow 0,\quad \quad \beta _i,\alpha _i\rightarrow \infty ,\quad \quad b\,\alpha _i\equiv a_i, \quad b\,\beta _i\equiv b_i\quad {\text {finite}}, \end{aligned}$$

under which the divergence of the conformal blocks exponentiates [47] and the *z*-dependence becomes subleading:^3^

2.16 $$\begin{aligned} \begin{aligned}&\mathfrak {F}\bigg ( \begin{matrix} \alpha _{1} \\ \alpha _{\infty } \end{matrix} \, \beta _{n-3} \, \begin{matrix} \alpha _{2} \\ \, \end{matrix} \,\dots \, \beta _1\,\begin{matrix} \alpha _{n-2} \\ \, \end{matrix} \, \alpha _{0\theta } \, \begin{matrix} \alpha _{2,1} \\ \alpha _0\end{matrix};\frac{z_2}{z_1},\frac{z_3}{z_2}\dots ,\frac{z_{n-2}}{z_{n-3}},\frac{z}{z_{n-2}}\bigg )\\&\quad =\,z_1^{\Delta _{\infty }-\Delta _{\alpha _1}-\Delta _{\beta _{n-3}}} z_2^{\Delta _{\beta _{n-3}}-\Delta _{\alpha _2}-\Delta _{\beta _{n-4}}}\dots z_{n-2}^{\Delta _{\beta _1}-\Delta _{\alpha _{n-2}}-\Delta _{\alpha _{0\theta }}}z^{\frac{b^2+1}{2}+\theta b\alpha _0}\\&\qquad \times \exp \left( \frac{1}{b^2}\left( F\left( \frac{z_2}{z_1},\frac{z_3}{z_2}\cdots ,\frac{z_{n-2}}{z_{n-3}}\right) +\mathcal {O}(b^2)\right) \right) , \end{aligned} \end{aligned}$$

where

2.17 $$\begin{aligned} F\left( \frac{z_2}{z_1},\frac{z_3}{z_2}\cdots ,\frac{z_{n-2}}{z_{n-3}}\right) =F\left( \begin{matrix} a_{1} \\ a_\infty \end{matrix} \begin{matrix} b_{n-3} \\ \end{matrix} \dots \begin{matrix} b_{1} \\ \end{matrix} \begin{matrix} a_{n-2} \\ a_0\end{matrix};\frac{z_2}{z_1},\frac{z_3}{z_2}\cdots ,\frac{z_{n-2}}{z_{n-3}}\right) \end{aligned}$$

is the semiclassical *n*-point conformal block which is defined as

2.18 $$\begin{aligned} \begin{aligned}&\mathfrak {F}\left( \begin{matrix} \alpha _{1} \\ \alpha _{\infty }\end{matrix}\,\beta _{n-3}\,\dots \,\beta _1\begin{matrix} \alpha _{n-2} \\ \alpha _0\end{matrix};{z_2\over z_1},\frac{z_3}{z_2}\cdots ,\frac{z_{n-2}}{z_{n-3}}\right) \\&\quad =\,z_1^{\Delta _{\infty }-\Delta _{\alpha _1}-\Delta _{\beta _{n-3}}}z_2^{\Delta _{\beta _{n-3}}-\Delta _{\alpha _2}-\Delta _{\beta _{n-4}}}\dots z_{n-2}^{\Delta _{\beta _1}-\Delta _{\alpha _{n-2}}-\Delta _{\alpha _{0}}}{{\,\mathrm{\textrm{e}}\,}}^{\frac{1}{b^2}\left( F\left( {z_2\over z_1},\frac{z_3}{z_2}\cdots ,\frac{z_{n-2}}{z_{n-3}}\right) +\mathcal {O}(b^2)\right) }. \end{aligned} \end{aligned}$$

Since the divergent parts for (2.16) and (2.18) are the same, following the procedure outlined in [19] we cure the divergences of (2.16) by dividing it by (2.18) and we denote the resulting finite semiclassical block by^4^

2.19 $$\begin{aligned} \begin{aligned}&\mathcal {F}\bigg ( \begin{matrix} a_{1} \\ a_{\infty } \end{matrix} \, b_{n-3} \, \begin{matrix} a_{2} \\ \, \end{matrix} \,\dots \, b_1\,\begin{matrix} a_{n-2} \\ \, \end{matrix} \, a_{0\theta } \, \begin{matrix} a_{2,1} \\ a_0\end{matrix};\frac{z_2}{z_1},\frac{z_3}{z_2}\dots ,\frac{z_{n-2}}{z_{n-3}},\frac{z}{z_{n-2}}\bigg )\\&\quad =\lim _{b\rightarrow 0}\frac{\mathfrak {F}\bigg ( \begin{matrix} \alpha _{1} \\ \alpha _{\infty } \end{matrix} \, \beta _{n-3} \, \begin{matrix} \alpha _{2} \\ \, \end{matrix} \,\dots \, \beta _1\,\begin{matrix} \alpha _{n-2} \\ \, \end{matrix} \, \alpha _{0\theta } \, \begin{matrix} \alpha _{2,1} \\ \alpha _0\end{matrix};{z_2\over z_1},\frac{z_3}{z_2}\dots ,\frac{z_{n-2}}{z_{n-3}},\frac{z}{z_{n-2}}\bigg )}{\mathfrak {F}\left( \begin{matrix} \alpha _{1} \\ \alpha _{\infty }\end{matrix}\,\beta _{n-3}\,\dots \,\beta _1\begin{matrix} \alpha _{n-2} \\ \alpha _0\end{matrix};{z_2\over z_1},\frac{z_3}{z_2}\dots ,\frac{z_{n-2}}{z_{n-3}}\right) }\\&\quad =z_{n-2}^{-\theta a_0}\,z^{\frac{1}{2}+\theta a_0}\,\exp \left( -\frac{\theta }{2}\partial _{a_0}F\left( {z_2\over z_1},\frac{z_3}{z_2}\dots ,\frac{z_{n-2}}{z_{n-3}}\right) \right) \cdot \left( 1+\mathcal {O}\left( z\right) \right) . \end{aligned} \end{aligned}$$

In the semiclassical limit, the BPZ equation (2.4) becomes a second-order ordinary differential equation with regular singularities at $z=z_i$, i=0,⋯,n-1

2.20 $$\begin{aligned} \begin{aligned}&\left( \frac{\textrm{d}^2}{\textrm{d}z^2}+\sum _{i=0}^{n-2}\frac{\frac{1}{4}-a_{i}^2}{(z-z_i)^2}+\frac{\big (\frac{1}{4}-a_{\infty }^2\big )-\sum _{i=0}^{n-2}\left( \frac{1}{4}-a_{i}^2\right) }{z(z-1)}\right. \\&\quad \left. +\sum _{i=2}^{n-2}\frac{(z_i-1)\,u_i}{z(z-1)(z-z_i)}\right) \psi _{0,\theta }(z)=0, \end{aligned} \end{aligned}$$

where

2.21 $$\begin{aligned} u_i=\lim _{b\rightarrow 0}b^2\,z_i\,\partial _{z_i}\,\log \,\mathfrak {F}\left( \begin{matrix} \alpha _{1} \\ \alpha _{\infty }\end{matrix}\,\beta _{n-3}\,\dots \,\beta _1\begin{matrix} \alpha _{n-2} \\ \alpha _0\end{matrix};{z_2\over z_1},\frac{z_3}{z_2}\dots ,\frac{z_{n-2}}{z_{n-3}}\right) ~,\end{aligned}$$

and

2.22 $$\begin{aligned} \begin{aligned}\mathcal {F}\bigg ( \begin{matrix} a_{1} \\ a_{\infty } \end{matrix} \, b_{n-3} \, \begin{matrix} a_{2} \\ \, \end{matrix} \,\dots \, b_1\,\begin{matrix} a_{n-2} \\ \, \end{matrix} \, a_{0\theta } \, \begin{matrix} a_{2,1} \\ a_0\end{matrix};{z_2\over z_1},\frac{z_3}{z_2},\dots ,\frac{z_{n-2}}{z_{n-3}},\frac{z}{z_{n-2}}\bigg )~&\propto ~\psi _{0,\theta }(x),\end{aligned}\end{aligned}$$

where $\psi _{0,\theta }(x)=(z)^{{1\over 2}+\theta a_0}(1+\mathcal {O}(z))$, $\theta =\pm$ denote the Frobenius solutions around z=0.

Let us stress that the forms of (2.21) and (2.22) are affected by the specific choice and order of the punctures $z_i$, as well as the point at which the degenerate field $\Phi _{2,1}(z)$ is inserted because they specify how the OPEs are taken. Our choices lead to a basis of solutions centered around z=0. However, as discussed in Sect. 2.2, solutions around different singular points can also be achieved by alternative choices of comb diagrams.

As we discuss in Sect. 2.3, the semiclassical BPZ equations are quantum Seiberg–Witten curves and can be solved by using the Nekrasov–Shatashvili approach [2]. In this context, (2.21) is known as Matone relation [48–50], and the local solutions to (2.20) correspond to partition functions of specific 4d supersymmetric gauge theories coupled to 2d defects.

### Connection coefficients from crossing symmetry

Around each singular point $z_i$ of Eq. (2.20) we can find a basis of Frobenius solutions $\psi _{z_i,\pm }(z)$ such that

2.23 $$\begin{aligned} \psi _{z_i,\pm }(z)\sim (z-z_i)^{\frac{1}{2} \pm a_i}\left( 1+\mathcal {O}\left( z-z_i\right) \right) \,. \end{aligned}$$

as well as

2.24 $$\begin{aligned} \psi _{\infty ,\pm }=z^{\frac{1}{2}\mp a_{\infty }}\left[ 1+\mathcal {O}\left( \frac{1}{z}\right) \right] . \end{aligned}$$

The radius of convergence of (2.23) is determined by the distance to the next closest singularity. However, it is possible to analytically continue $\psi _{z_i,\pm }(z)$ to a different puncture $z_j$ and express $\psi _{z_j,\pm }(z)$ as

2.25 $$\begin{aligned} \psi _{z_j,\pm }(z)=\mathcal {C}^{z_i,+}_{z_j,\pm }\psi _{z_i,+}(z)+\mathcal {C}^{z_i,-}_{z_j,\pm }\psi _{z_i,-}(z),\end{aligned}$$

where the coefficients $\mathcal {C}^{z_j,\pm }_{z_i,\pm }$ are known as the connection coefficients.

As pointed out in [19], an efficient approach to derive closed-form expressions for these coefficients involves the use of crossing symmetry in the underlying Liouville CFT. In [19], the case of the Heun equation (four regular singular points), was thoroughly analyzed. In this paper, we extend these results to scenarios in which the ODE has more than four singularities, with special attention to the case of five singular points.

To study the connection coefficients in (2.25), we use the Frobenius expansions of $$\psi _{z_i,\theta }(z)$$ in (2.23) corresponding to the semiclassical limit of a particular conformal block of the form $$\mathfrak {F}\left( \begin{matrix} \\ \alpha _{j} \end{matrix}\cdots \begin{matrix} \alpha _{1} \\ \hspace{0.1cm} \end{matrix}\cdots \alpha _{i\theta }\begin{matrix} \alpha _{2,1} \\ \alpha _{i} \end{matrix}; \cdots \right) $$, where the OPE of Φ(2,1)(z) and $$V_{\alpha _i}(z_i)$$ is given in (2.6). Such block can be represented schematically as the following comb diagram2.26This configuration can be obtained by imposing the Möbius transform for the $$PGL(2,\mathbb {C})$$ symmetry starting from (2.12) up to a normalization which comes from the transformation of the primary fields under $x\to x^{\prime }$ as

2.27 $$\begin{aligned} V_{\alpha }(x)~\rightarrow ~ V'_{\alpha }(x')=\left( \frac{\textrm{d}x'}{\textrm{d}x}\right) ^{-\Delta _{\alpha }}V_{\alpha }(x)~.\end{aligned}$$

In this way, one has a direct map between Frobenius solutions and conformal blocks. Similarly, we use the Frobenius expansions of $\psi _{z_j,\theta }(z)$ corresponding to the semiclassical limit of a particular conformal block of the form2.28We remark that when there are six or more insertions, a new structure of diagram can arise, which cannot be represented as a comb-like one. The first example of this kind arises when there are six punctures, which are arranged in three pairs of nearby insertions. In the topological string and gauge theory literature, these are associated with nonlinear (or Sicilian) quivers (see [51–53] for a discussion). We will see an example of this kind (in the presence of a degenerate insertion) in Sect. 3.3.

Then, we want to relate the solutions around $z_i$, i.e., $\psi _{z_i,\pm }(z)$, to the solutions around $z_j$, i.e.  $\psi _{z_j,\pm }(z)$. In the language of CFT, this implies that our task is to examine the effects of exchanging the positions of $\alpha _{2,1}$ and the punctures in between $z_i$ and $z_j$. This effect can be obtained by the product of the factors corresponding to the swapping of $\alpha _{2,1}$ and $\alpha _k$. Each single step from2.29to2.30is a local effect analogous to the one of hypergeometric functions. To be more precise, the comb diagrams (2.13) and (2.29) are related by crossing symmetry as^5^

2.31 $$\begin{aligned}&\int \prod _i\textrm{d}\beta _i\ \sum _{\theta =\pm }C^{\beta _{m\theta }}_{\alpha _{2,1}\beta _m}C_{\alpha _{k}\beta _{m\theta }}^{\beta _l}\,\bigg |\mathfrak {F}\left( \cdots \beta _l\begin{matrix} \alpha _{k} \\ \, \end{matrix} \,\beta _{m\theta } \, \begin{matrix} \alpha _{2,1} \\ \, \end{matrix}\beta _m\cdots ; \cdots \right) \bigg |^2\nonumber \\&\quad = \int \prod _i\textrm{d}\beta _i\ \sum _{\theta '=\pm }C^{\beta _{l\theta '}}_{\alpha _k\beta _m}C_{\alpha _{2,1}\beta _{l\theta '}}^{\beta _l}\,\bigg |\mathfrak {F}\left( \cdots \beta _l\begin{matrix} \alpha _{2,1} \\ \, \end{matrix} \,\beta _{l\theta '} \, \begin{matrix} \alpha _{k} \\ \, \end{matrix}\beta _m\cdots ; \cdots \right) \bigg |^2,\end{aligned}$$

which is satisfied if

2.32 $$\begin{aligned}&\sum _{\theta =\pm }C^{\beta _{m\theta }}_{\alpha _{2,1}\beta _m}C_{\alpha _{k}\beta _{m\theta }}^{\beta _l}\,\bigg |\mathfrak {F}\left( \cdots \beta _l\begin{matrix} \alpha _{k} \\ \, \end{matrix} \,\beta _{m\theta } \, \begin{matrix} \alpha _{2,1} \\ \, \end{matrix}\beta _m\cdots ; \cdots \right) \bigg |^2\nonumber \\&\quad =\sum _{\theta '=\pm }C^{\beta _{l\theta '}}_{\alpha _k\beta _m}C_{\alpha _{2,1}\beta _{l\theta '}}^{\beta _l}\,\bigg |\mathfrak {F}\left( \cdots \beta _l\begin{matrix} \alpha _{2,1} \\ \, \end{matrix} \,\beta _{l\theta '} \, \begin{matrix} \alpha _{k} \\ \, \end{matrix}\beta _m\cdots ; \cdots \right) \bigg |^2,\end{aligned}$$

and we also refer to (2.32) as crossing symmetry. Parallel to [19], we can check that (2.32) is satisfied by

2.33 $$\begin{aligned} \mathfrak {F}\left( \cdots \beta _l\begin{matrix} \alpha _{k} \\ \, \end{matrix} \,\beta _{m\theta } \, \begin{matrix} \alpha _{2,1} \\ \, \end{matrix}\beta _m\cdots ; \cdots \right)  &   =\sum _{\theta '=\pm }{\mathrm e}^{\textrm{i}\wp _{\theta '}}\mathcal {M}_{\theta \theta '}(b\,\beta _m,b\,\beta _l;b\,\alpha _k)\mathfrak {F}\nonumber \\  &   \quad \left( \cdots \beta _l\begin{matrix} \alpha _{2,1} \\ \, \end{matrix} \,\beta _{l\theta '} \, \begin{matrix} \alpha _{k} \\ \, \end{matrix}\beta _m\cdots ; \cdots \right) \end{aligned}$$

if

2.34 $$\begin{aligned} \mathcal {M}_{\theta \theta '}(a_1,a_2;a_3) = \frac{\Gamma (-2\theta 'a_2)\Gamma (1+2\theta a_1)}{\Gamma \left( \frac{1}{2}+\theta a_1-\theta ' a_2 + a_3\right) \Gamma \left( \frac{1}{2}+\theta a_1-\theta ' a_2 - a_3\right) },\quad \theta ,\theta '=\pm , \end{aligned}$$

where ${\mathrm e}^{\textrm{i}\wp _{\pm }}$ is a phase factor, which can be fixed by looking at the leading power in the *z*-expansion of the OPE. We will fix this phase in concrete examples below. The above formulae, as well as the ones we will compute below, hold for generic values of the indicial parameters. In principle, one should assume $2a_i \notin \mathbb {Z}$ in order to avoid logarithmic singularities. However, it has been verified in numerous examples in [54] that, if ones take the limit $2a_i \rightarrow \mathbb {Z}$ carefully, the formulas above continue to hold.

As an example, let us take the special case where we connect the points at 0 and ∞ and we suppose that the singularity structure can be represented by a comb-like diagram. By applying the connection formula repeatedly, we find (up to phases)

2.35 $$\begin{aligned} \begin{aligned}&\mathfrak {F}\bigg ( \begin{matrix} \alpha _{{1}} \\ \alpha _{\infty } \end{matrix} \, \beta _{n-3} \, \begin{matrix} \alpha _{{2}} \\ \, \end{matrix} \,\dots \, \beta _1\,\begin{matrix} \alpha _{n-2} \\ \, \end{matrix} \, \alpha _{0\theta } \, \begin{matrix} \alpha _{2,1} \\ \alpha _0\end{matrix};\frac{z_{2}}{z_{1}},\frac{z_{3}}{z_{2}},\dots ,\frac{z}{z_{n-2}}\bigg )\\&\quad =\sum _{\theta _2,\dots ,\theta _{n-1}=\pm }\mathcal {M}_{\theta \theta _2}\left( b\alpha _0,b\beta _1;b\alpha _{n-2}\right) \mathcal {M}_{(-\theta _2)\theta _3}\left( b\beta _1,b\beta _2;b\alpha _{n-3}\right) \\&\qquad \dots \mathcal {M}_{(-\theta _{n-3})\theta _{n-2}}\left( b\beta _{n-4},b\beta _{n-3};b\alpha _{{2}}\right) \\&\qquad \times \mathcal {M}_{(-\theta _{n-2})\theta _{n-1}}\left( b\beta _{n-3},b\alpha _{\infty };b\alpha _{{1}}\right) (-1)^{\Delta _0+\Delta _\infty }\, \\&\qquad \times \prod _{\begin{array}{c} k=1 \end{array}}^{n-2} (-z_k^{-2})^{\Delta _{z_k}}(-z^{-2})^{\Delta _{2,1}}\,\mathfrak {F}\bigg ( \begin{matrix} \alpha _{n-2} \\ \alpha _{0} \end{matrix} \, \beta _{1\theta _2} \, \begin{matrix} \alpha _{{n-3}} \\ \, \end{matrix} \,\dots \, \beta _{(n-3)\theta _{n-2}}\,\begin{matrix} \alpha _{{1}} \\ \, \end{matrix} \, \alpha _{\infty \theta _{n-1}} \, \\&\qquad \begin{matrix} \alpha _{2,1} \\ \alpha _{\infty }\end{matrix};\frac{z_{n-2}}{z_{n-3}},\dots ,\frac{z_{2}}{z_1},\frac{z_{1}}{z}\bigg ). \end{aligned} \end{aligned}$$

In the semiclassical limit b→0, using (2.16), we relate the latter conformal block to the one without the shifts in the intermediate momenta:

2.36 $$\begin{aligned} \begin{aligned}&\mathfrak {F}\bigg ( \begin{matrix} \alpha _{n-2} \\ \alpha _{0} \end{matrix} \, \beta _{1\theta _2} \, \begin{matrix} \alpha _{{n-3}} \\ \, \end{matrix} \,\dots \, \beta _{(n-3)\theta _{n-2}}\,\begin{matrix} \alpha _{{1}} \\ \, \end{matrix} \, \alpha _{\infty \theta _{n-1}} \, \begin{matrix} \alpha _{2,1} \\ \alpha _{\infty }\end{matrix};\frac{z_{n-2}}{z_{n-3}},\dots ,\frac{z_{2}}{z_1},\frac{z_{1}}{z}\bigg )\\&=\left( \frac{z_{2}}{z_{1}}\right) ^{\theta _{n-2}b_{n-3}}\dots \left( \frac{z_{n-2}}{z_{n-3}}\right) ^{\theta _2b_{1}}\exp \left( -\sum _{k=1}^{n-3}\frac{\theta _{k+1}}{2}\partial _{b_k}F\left( \frac{z_{n-2}}{z_{n-3}},\dots ,\frac{z_{2}}{z_1}\right) +\mathcal {O}(b)\right) \\&\ \times \mathfrak {F}\bigg ( \begin{matrix} \alpha _{n-2} \\ \alpha _{0} \end{matrix} \, \beta _{1} \, \begin{matrix} \alpha _{{n-3}} \\ \, \end{matrix} \,\dots \, \beta _{n-3}\,\begin{matrix} \alpha _{{1}} \\ \, \end{matrix} \, \alpha _{\infty \theta _{n-1}} \, \begin{matrix} \alpha _{2,1} \\ \alpha _{\infty }\end{matrix};\frac{z_{n-2}}{z_{n-3}},\dots ,\frac{z_{2}}{z_1},\frac{z_{1}}{z}\bigg ). \end{aligned} \end{aligned}$$

Therefore, if we take the semiclassical limit

2.37 $$\begin{aligned} b\rightarrow 0,\quad \beta _i, \alpha _i\rightarrow \infty ,\quad b\alpha _i\equiv a_i\quad b\beta _i\equiv b_i\ {\text {finite}}, \end{aligned}$$

of the connection formula (2.35), we find (up to phases)

2.38 $$\begin{aligned} \begin{aligned}&\mathcal {F}\bigg ( \begin{matrix} a_{{1}} \\ a_{\infty } \end{matrix} \, b_{n-3} \, \begin{matrix} a_{{2}} \\ \, \end{matrix} \,\dots \, b_1\,\begin{matrix} a_{n-2} \\ \, \end{matrix} \, a_{0\theta } \, \begin{matrix} a_{2,1} \\ a_0\end{matrix};\frac{z_{2}}{z_{1}},\frac{z_{3}}{z_{2}},\dots ,\frac{z}{z_{n-2}}\bigg )\\&\quad =\sum _{\theta _2,\dots ,\theta _{n-1}=\pm }\mathcal {M}_{\theta _1\theta _2}\left( a_0,b_1;a_{n-2}\right) \mathcal {M}_{(-\theta _2)\theta _3}\left( b_1,b_2;a_{n-3}\right) \\&\qquad \dots \mathcal {M}_{(-\theta _{n-3})\theta _{n-2}}\left( b_{n-4},b_{n-3};a_{{2}}\right) \\&\qquad \times \mathcal {M}_{(-\theta _{n-2})\theta _{n-1}}\left( b_{n-3},a_{\infty };a_{1}\right) \left( \frac{z_{2}}{z_{1}}\right) ^{\theta _{n-2}b_{n-3}}\\&\qquad \dots \left( \frac{z_{n-2}}{z_{n-3}}\right) ^{\theta _2b_{1}}{{\,\mathrm{\textrm{e}}\,}}^{-\sum _{k=1}^{n-3}\frac{\theta _{k+1}}{2}\partial _{b_k}F\left( \frac{z_{n-2}}{z_{n-3}},\dots ,\frac{z_{2}}{z_1}\right) }\\&\qquad \times \textrm{i}\, z\,\mathcal {F}\bigg ( \begin{matrix} a_{n-2} \\ a_{0} \end{matrix} \, b_{1} \, \begin{matrix} a_{{n-3}} \\ \, \end{matrix} \,\dots \, b_{n-3}\,\begin{matrix} a_{{1}} \\ \, \end{matrix} \, a_{\infty \theta _{n-1}} \, \begin{matrix} \alpha _{2,1} \\ a_{\infty }\end{matrix};\frac{z_{n-2}}{z_{n-3}},\dots ,\frac{z_{2}}{z_1},\frac{z_{1}}{z}\bigg ). \end{aligned} \end{aligned}$$

To fully solve the connection problem, we also want to find the proportionality factor between the blocks and the Frobenius solutions. From (2.19), we have

2.39 $$\begin{aligned}  &   \mathcal {F}\bigg ( \begin{matrix} a_{{1}} \\ a_{\infty } \end{matrix} \, b_{n-3} \, \begin{matrix} a_{{2}} \\ \, \end{matrix} \,\dots \, b_1\,\begin{matrix} a_{n-2} \\ \, \end{matrix} \, a_{0\theta } \, \begin{matrix} a_{2,1} \\ a_0\end{matrix};\frac{z_{2}}{z_{1}},\frac{z_{3}}{z_{2}},\dots ,\frac{z}{z_{n-2}}\bigg ) \nonumber \\  &   \quad = z_{n-2}^{-\theta a_0}{{\,\mathrm{\textrm{e}}\,}}^{-{\theta \over 2} \partial _{a_0} F\left( \frac{z_{2}}{z_{1}},\frac{z_{3}}{z_{2}},\dots ,\frac{z_{n-2}}{z_{n-3}}\right) }\psi _{0,\theta } \end{aligned}$$

2.40 $$\begin{aligned}  &   \,z\,\mathcal {F}\bigg ( \begin{matrix} a_{n-2} \\ a_{0} \end{matrix} \, b_{1} \, \begin{matrix} a_{{n-3}} \\ \, \end{matrix} \,\dots \, b_{n-3}\,\begin{matrix} a_{{1}} \\ \, \end{matrix} \, a_{\infty \theta } \, \begin{matrix} \alpha _{2,1} \\ a_{\infty }\end{matrix};\frac{z_{n-2}}{z_{n-3}},\dots ,\frac{z_{2}}{z_1},\frac{z_{1}}{z}\bigg ) \nonumber \\  &   \quad =z_{1}^{-\theta a_{\infty }}{{\,\mathrm{\textrm{e}}\,}}^{-{\theta \over 2} \partial _{a_{\infty }} F\left( \frac{z_{2}}{z_{1}},\frac{z_{3}}{z_{2}},\dots ,\frac{z_{n-2}}{z_{n-3}}\right) } \psi _{\infty ,\theta }~.\end{aligned}$$

In this general discussion, we only consider comb-like configurations of the punctures. As already mentioned, when dealing with differential equations with 5 or more singularities, the situation can be more complicated because of a possibly more involved decomposition of the singularity structure. The analysis of the connection steps remains unchanged, and the final result is still a concatenation of hypergeometric-like connections, but the expansion parameters of the conformal blocks may differ.

### Solving BPZ equations via gauge theory

The connection between classical integrable systems and Seiberg–Witten theory has been known for some time, see [55] for a review and references. However, the extension of this relation to the quantum level was not immediately obvious. Nekrasov and Shatashvili addressed this in [2], where they found that transitioning to quantum integrable systems involves activating one of the two parameters in the Ω-background [56–58]. Among various aspects, this result had a notable implication: it provided a systematic framework for solving the spectral theory of a class of differential equations known as four-dimensional quantum Seiberg–Witten curves. The key of this approach lies in the NS functions, which serve as building blocks to construct such solutions.

Since the BPZ equations in the semiclassical limit are specific examples of four-dimensional quantum Seiberg–Witten curves, the work of Nekrasov and Shatashvili naturally finds interpretation within Liouville CFT via the AGT correspondence [21]. Although viewing the problem from the perspective of Liouville CFT is not strictly necessary, it can sometimes offer an alternative and efficient approach to performing computations.

For the purpose of this work, we focus on four-dimensional $\mathcal {N}=2$ superconformal linear quiver gauge theories with gauge group $SU(2)^{n-3}$ in the Ω background which have class $\mathcal {S}$ descriptions [40]. A theory of class $\mathcal {S}$ is specified by the following data: a 6d $\mathcal {N}=(2,0)$ theory specified by a Lie algebra $\mathfrak {g}$ of ADE type; a punctured Riemann surface *C*, where the punctures are at ${z_i}$’s; a codimension-2 defect for each puncture ${z_i}$. With the above data given, a class $\mathcal {S}$ theory is defined as the 4d theory obtained by compactifying the 6d $\mathcal {N}=(2,0)$ theory with defects on the punctured Riemann surface *C*. For $\mathfrak {g}={\text {A}}_1$, the Seiberg–Witten curve has the form

2.41 $$\begin{aligned} \Sigma =\{\lambda ^2-P(z)\textrm{d}z^2=0\}\subset T^*C, \end{aligned}$$

where z∈C and $P(z)\textrm{d}z^2$ is a meromorphic quadratic differential.

Note that, in general, if we have more than five punctures, there are different realizations of the $\mathcal {N}=2$ theory as a weakly coupled generalized quiver [40]. Here, we will consider the case of linear quiver gauge theory.

An $SU(2)^{n-3}$ linear quiver gauge theory can be represented by (see for instance [59] and reference therein)

**Figure:**
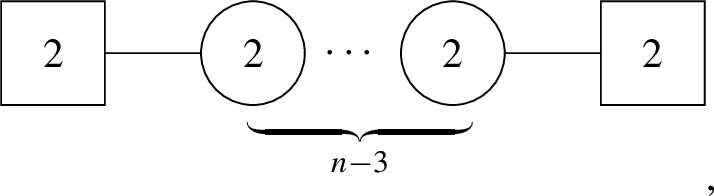

where the n-3 circles represent the n-3 *SU*(2) gauge groups. There are n-3 vector multiplets of the $SU(2)^{n-3}$ gauge groups.

Besides the vector multiplets of the $SU(2)^{n-3}$ gauge group, such a quiver theory contains the following matter superfields

- n-4 bifundamental hypermultiplets for $SU(2)_{j-1}\times SU(2)_{j}$ whose masses are given by the parameters $a_{j}$, j=2,⋯,n-3.
- 2 fundamental hypermultiplets for $SU(2)_{n-3}$, represented by the square boxes on the right, with masses^6^
  2.42 $$\begin{aligned} m_1=a_0-a_{n-2},\quad m_2=a_0+a_{n-2}, \end{aligned}$$
- 2 antifundamental hypermultiplets, represented by the square boxes on the left, for $SU(2)_1$, with masses
  2.43 $$\begin{aligned} m_3=a_{\infty }-a_{1},\quad m_4=a_{\infty }+a_{1}. \end{aligned}$$

For each gauge factor $SU(2)_i$, the corresponding Yang–Mills coupling constant $g_i$ and the theta-angles $\theta _i$, i=1,⋯,n-3, are combined into the complexified gauge coupling

2.44 $$\begin{aligned} \tau _i=\frac{\theta _i}{2\pi }+\textrm{i}\,\frac{4\pi }{g_i^2}. \end{aligned}$$

The vacuum expectation value of the adjoint scalar in the vector multiplet for each gauge factor *SU*(2) is $\vec {b}_i=\textrm{diag}(b_{i},-b_{i})$.

We can turn on the Ω background deformation of the theory which introduces two more parameters $\epsilon _1$ and $\epsilon _2$ corresponding to the rotations on two perpendicular planes. In this case, the partition function of the 4d supersymmetric gauge theory can be obtained by the localization method [56–58, 60]. The result is known as the Nekrasov partition function, which is defined as a series in the exponential of the gauge coupling constants $\tau _i$, while each coefficient is a meromorphic function of $b_i$’s and $m_i$’s. An example can be found in (A.3).

We can further insert a surface defect into the theory [8–15, 41, 61, 62]. Since we restrict ourselves to the theories of class $\mathcal {S}$, there exists a canonical surface defect whose moduli space is the same Riemann surface *C*. Without loss of generality, we assume that the surface defect corresponds to z∈C such that $0<z<z_1<\cdots z_n<\infty$. The Nekrasov partition function with the insertion of a surface defect has one more expansion parameter, $\frac{z}{z_1}$. An example can be found in (B.1).

Recall that we have two Ω background parameters $\epsilon _1$ and $\epsilon _2$. There is an interesting limit where, if we assume that $\epsilon _1$ corresponds to the rotation along the canonical surface defect, we take $\epsilon _2\rightarrow 0$ while keeping $\epsilon _1$ finite. This is known as the NS limit. In this particular deformation, the Seiberg–Witten curve is quantized into an oper

2.45 $$\begin{aligned} \nabla =\partial _z+\epsilon _1^{-1} \begin{pmatrix} 0 & \quad -P(z)\\ 1 & \quad 0\end{pmatrix}.\end{aligned}$$

Setting $\epsilon _1=1$, such an oper can be recasted into a second-order ODE

2.46 $$\begin{aligned} \begin{aligned} \left( \frac{{\textrm{d}}^2}{{\textrm{d}z}^2}+P(z)\right) \psi (z)=0, \end{aligned} \end{aligned}$$

whose spectral theory is fully determined by the NS functions.

The above gauge theory is related to conformal field theory via the famous AGT correspondence, see [63] for a review. The AGT correspondence relates Liouville and Toda CFTs to 4d $\mathcal {N}=2$ theories which have class $\mathcal {S}$ realizations [14, 20, 21] where conformal blocks are identified with Nekrasov partition functions. For $SU(2)^{n-3}$ quiver theories, the masses $a_i$’s are mapped to the momenta of regular singularities via $b\alpha _i\equiv a_i$, which are denoted by the same $\alpha _i$’s for the reason of this identification. And the scalar v.e.v $b_i$ are mapped to the momenta on internal legs through $b\beta _i\equiv b_i$. The gauge coupling constants are related to the positions of the punctures as

2.47 $$\begin{aligned} \exp (2\pi \textrm{i}\tau _i)=\frac{z_i}{z_{i+1}} ,\end{aligned}$$

where we assume $0<z_1<\cdots z_n<\infty$.

In the dictionary relating the two sides, the parametrization *b* of the central charge *c* can be related to the Ω background parameters $\epsilon _i$ by

2.48 $$\begin{aligned} b=\sqrt{\frac{\epsilon _2}{\epsilon _1}}. \end{aligned}$$

In the convention of this paper, we take $\epsilon _1=1$ and $\epsilon _2=b^2$. The semiclassical limit of the 2d CFT is therefore mapped to the NS limit of the supersymmetric quiver gauge theories. The second-order ODE (2.46) we mentioned above gives exactly the semiclassical limit of the corresponding BPZ equation. The Nekrasov partition function, incorporating a surface defect in the 4d/2d coupled gauge theory system, is mapped to the conformal blocks arising in the decomposition of the (n+1)-point correlation function (2.5), which involves n primary fields and a degenerate field $\Phi _{2,1}(z)$.

## Example: Fuchsian equation with five singularities

In this section, we focus on the case of a second-order differential equations with five regular singular points at $z=\{0,1,t,q,\infty \}$:

3.1 $$\begin{aligned} \begin{aligned}&\left( \frac{\textrm{d}}{\textrm{d}z^2}+\sum _{i=0,1,t,q}\frac{\frac{1}{4}-a_{i}^2}{(z-z_i)^2}+\frac{\frac{1}{4}-a_{\infty }^2-\sum _{i=0,1,t,q}\big (\frac{1}{4}-a_{i}^2\big )}{z(z-1)}\right. \\&\quad \left. +\frac{(t-1)\,u_t}{z(z-1)(z-t)}+\frac{\left( q-1\right) \,u_{q}}{z(z-1)\left( z-q\right) }\right) \psi (z)=0. \end{aligned} \end{aligned}$$

**Fig. 1:**
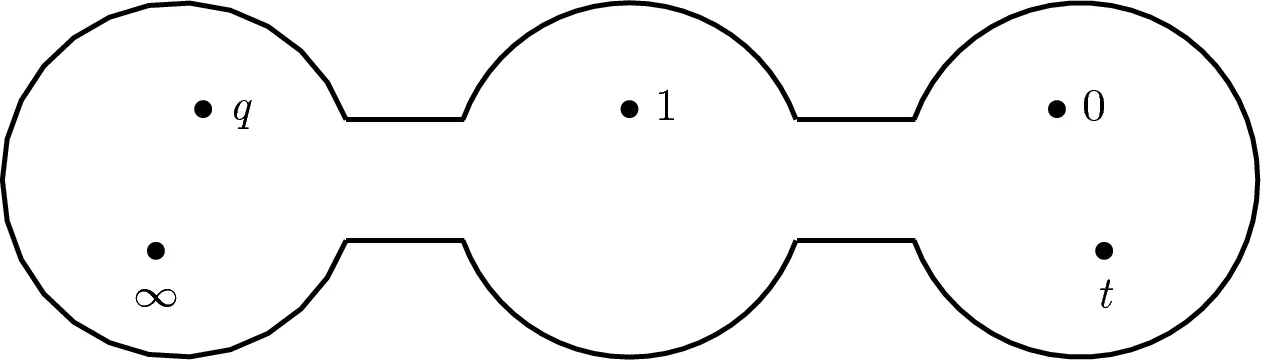
Sphere decomposition

We examine the regime for the positions of the singularities t and q illustrated in Fig. 1, specifically 0<|t|<1<|q|<∞.

### The gauge theory

The Riemann surface where *z* of the differential equation (3.1) lives can be described as a sphere with 5 punctures. This curve admits a weakly-coupled description as a $\mathcal {N}=2$ superconformal linear quiver gauge theory with gauge group SU(2)×SU(2), see Sect. 2.3. In this section, the masses of the fundamental hypermultiplets are denoted by $m_{1,2}=a_t \pm a_0$ and $m_{3,4}=a_q \pm a_{\infty }$, while $a_1$ represents the mass of the matter in the bifundamental. There are two parameters, that we denote with $b_1$ and $b_2$, which represent the v.e.v. of the scalars in the $\mathcal {N}=2$ vector multiplets. These are related to the complex moduli $u_t$ and $u_q$ appearing in the potential of (3.1) by the Matone relations (2.21), whose form depends on the decomposition of the five-punctured sphere.

In the case where the punctures are distributed as in Fig. 1, we have $z_1=0, z_2=t, z_3=1, z_4=q, z_5=\infty$. Using this in (2.21) gives

3.2 $$\begin{aligned} u_{t}&=\lim _{b\rightarrow 0}b^2\,t\,\partial _{t}\,\log \,\mathfrak {F}\left( \begin{matrix} \alpha _{q} \\ \alpha _{\infty }\end{matrix}\,\beta _{2}\, \begin{matrix} \alpha _{1} \\ ~\end{matrix}\, \beta _1\, \begin{matrix} \alpha _{t} \\ \alpha _0\end{matrix};\frac{1}{q}, t\right) =-\frac{1}{4}- b_1^2 + a_0^2 + a_t^2+t\frac{\partial F\left( {1\over q},t\right) }{\partial t},\end{aligned}$$

3.3 $$\begin{aligned} u_{q}&=\lim _{b\rightarrow 0}b^2\,q\,\partial _{q}\,\log \,\mathfrak {F}\left( \begin{matrix} \alpha _{q} \\ \alpha _{\infty }\end{matrix}\,\beta _{2}\, \begin{matrix} \alpha _{1} \\ ~\end{matrix}\, \beta _1\, \begin{matrix} \alpha _{t} \\ \alpha _0\end{matrix};\frac{1}{q}, t\right) =-\frac{1}{4}+b_2^2-a_{\infty }^2+a_{q}^2+q\frac{\partial F\left( {1\over q},t\right) }{\partial q}, \end{aligned}$$

where *F* is the Nekrasov–Shatashvili function, which we add more details about in “Appendix A”. The instanton expansions of the v.e.v. parameters in this case are of the following forms:

3.4 $$\begin{aligned} \begin{aligned} b_1&=\sum _{i,j\ge 0}b_1^{(i,j)}\,t^i\,\frac{1}{q^j},\quad&{\text {with}}\quad b_1^{(i,j)}=0\quad {\text {for}}\quad i<j,\\ b_2&=\sum _{i,j\ge 0}b_2^{(i,j)}\,t^i\,\frac{1}{q^j},\quad&{\text {with}}\quad b_2^{(i,j)}=0\quad {\text {for}}\quad j<i. \end{aligned} \end{aligned}$$

We will say that the *K*th instanton expansion of $b_i$, i=1,2, is determined if all the coefficients $b_i^{(m,n)}$ with m+n=K are known. The Matone relations can be inverted order by order in the two instanton parameters. In particular, for each K>0, the *K*th instanton expansion of $b_1$ requires the (K-1)th instanton expansion of $b_2$, and vice versa.

The first few coefficients in the expansions (3.4) are given by

3.5 $$\begin{aligned} b_1^{(0,0)}&=\sqrt{-\frac{1}{4}-u_{t}+a_{0}^2+a_{t}^2},\nonumber \\ b_1^{(1,0)}&=\frac{\left( 1-4 a_{t}^2+2 u_{t}\right) \left( 4 a_{0}^2+4 a_{1}^2-4 a_{\infty }^2+4 a_{t}^2+4 a_{q}^2-4 u_{t}-4 u_{q}-3\right) }{8 \sqrt{4 a_{0}^2+4 a_{t}^2-4 u_{t}-1} \left( 2 a_{0}^2+2 a_{t}^2-2 u_{t}-1\right) },\nonumber \\ b_2^{(0,0)}&=\sqrt{\frac{1}{4}+u_{q}+a_{\infty }^2-a_{q}^2},\nonumber \\ b_2^{(0,1)}&=-\frac{u_{q} \left( -4 a_{0}^2+4 a_{1}^2+4 a_{\infty }^2-4 a_{t}^2-4 a_{q}^2+4 u_{t}+4 u_{q}+1\right) }{8 \left( a_{\infty }^2-a_{q}^2+u_{q}\right) \sqrt{4 a_{\infty }^2-4 a_{q}^2+4 u_{q}+1}}. \end{aligned}$$

To be more precise, the inversion of the Matone relation would lead to two solutions for each v.e.v. parameter, differing for an overall sign; in the above results, we chose the branch with the plus sign in front of the canonical branch of square root. We computed the inverted Matone relations up to 4 instantons, and we noticed the triangular structure of the expansion of the v.e.v. parameters as in Eq. 3.4. We do not have a proof of the triangular structure of these expansions. However, we found that it holds at all analytically computed orders (up to i+j=5) and that it is in good agreement with the numerical results presented in later sections. It would be interesting to gain a better understanding of this structure from a CFT perspective.

In the forthcoming sections, we apply the strategy outlined in Sect. 2.2 to some specific examples.

### Connection problem I

We consider the configuration in Fig. 1. As a warm-up, we compute the connection coefficients between 0 and *t*. This case closely resembles that of the Heun equation as discussed in [54, 64, 65]. We define the Frobenius solutions around these points as

3.6 $$\begin{aligned} \begin{aligned} \psi _{t,\pm }(z)&=(z-t)^{\frac{1}{2}\pm a_t}\left( 1+\sum _{j\ge 1}c^{t,-}_{j}(z-t)^j\right) ,\\ \psi _{0,\pm }(z)&=z^{\frac{1}{2}\pm a_0}\left( 1+\sum _{j\ge 1}c^{0,\pm }_{j}z^j\right) . \end{aligned} \end{aligned}$$

Using the gauge theoretic expression of the conformal blocks (B.2) given in “Appendix B”, we find

3.7 $$\begin{aligned} \psi _{0,\theta }(z)=t^{\theta a_{0} }{\mathrm e}^{\frac{1}{2}\theta \partial _{a_0}F\left( \frac{1}{q} , t\right) }\mathcal {F}\left( \begin{matrix}a_q\\ a_{\infty }\end{matrix}\,b_2\,\begin{matrix}a_1\\ {}\end{matrix}\,b_1\,\begin{matrix} a_t\\ {}\end{matrix}\,a_{0\theta }\,\begin{matrix}a_{2,1}\\ a_{0}\end{matrix};\frac{1}{q},t,\frac{z}{t}\right) . \end{aligned}$$

Likewise, the Frobenius solutions around *t* are

3.8 $$\begin{aligned} \psi _{t,\theta }(z)= &   \left( \frac{1}{1-t}\right) ^{-\frac{1}{2}-\theta a_t}\left( \frac{t}{t-1}\right) ^{\theta a_{t} }{{\,\mathrm{\textrm{e}}\,}}^{\frac{\theta }{2}\partial _{a_t}F\left( {1-t\over q-t},\frac{t}{t-1}\right) }\,\nonumber \\  &   \mathcal {F}\left( \begin{matrix} a_{q} \\ a_{\infty } \end{matrix} \, b_2 \, \begin{matrix} a_1 \\ \, \end{matrix} \,b_1 \, \begin{matrix} a_0 \\ \, \end{matrix} \,a_{t\theta } \, \begin{matrix} a_{2,1} \\ a_t \end{matrix}; \frac{1-t}{q-t}, \frac{t}{t-1}, \frac{-z+t}{t} \right) ~.\end{aligned}$$

By using (2.18) and (2.27), we have

3.9 $$\begin{aligned} \left( \frac{t}{t-1}\right) ^{-\theta a_{t} }{{\,\mathrm{\textrm{e}}\,}}^{-\frac{\theta }{2}\partial _{a_t}F\left( {1-t\over q-t},\frac{t}{t-1}\right) } ={\mathrm e}^{-\frac{1}{2}\theta \partial _{a_t}\lim \limits _{b\rightarrow 0}b^2\log \mathfrak {F}\left( \begin{matrix}\alpha _q\\ \alpha _{\infty }\end{matrix}\,\beta _2\,\begin{matrix}\alpha _1\\ {}\end{matrix}\,\beta _1\,\begin{matrix}\alpha _0\\ \alpha _{t}\end{matrix};{1-t\over q-t},\frac{t}{t-1}\right) }. \end{aligned}$$

Since^7^

3.10 $$\begin{aligned}&\partial _{a_t}\lim \limits _{b\rightarrow 0}b^2\log \mathfrak {F}\left( \begin{matrix}\alpha _q\\ \alpha _{\infty }\end{matrix}\,\beta _2\,\begin{matrix}\alpha _1\\ {}\end{matrix}\,\beta _1\,\begin{matrix}\alpha _t\\ \alpha _{0}\end{matrix};{1\over q},t\right) \end{aligned}$$

3.11 $$\begin{aligned}&=\partial _{a_t}\lim \limits _{b\rightarrow 0}b^2\left( \log \mathfrak {F}\left( \begin{matrix}\alpha _q\\ \alpha _{\infty }\end{matrix}\,\beta _2\,\begin{matrix}\alpha _1\\ {}\end{matrix}\,\beta _1\,\begin{matrix}\alpha _0\\ \alpha _{t}\end{matrix};{1-t\over q-t},\frac{t}{t-1}\right) \left( \frac{1}{1-t}\right) ^{\Delta _0+\Delta _1+\Delta _t+\Delta _q+\Delta _\infty }\right) ~, \end{aligned}$$

we have

3.12 $$\begin{aligned} \left( \frac{t}{(t-1)}\right) ^{-\theta a_{t} }{{\,\mathrm{\textrm{e}}\,}}^{-\frac{\theta }{2}\partial _{a_t}F\left( {1-t\over q-t},\frac{t}{t-1}\right) }=t^{-\theta a_t}{{\,\mathrm{\textrm{e}}\,}}^{-\frac{\theta }{2}\partial _{a_t}F\left( {1\over q},t\right) }\left( \frac{1}{1-t}\right) ^{-\theta a_t}. \end{aligned}$$

Hence,

3.13 $$\begin{aligned} \begin{aligned}\psi _{t,\theta }(z)&= \left( \frac{1}{1-t}\right) ^{-\frac{1}{2}-\theta a_t}t^{\theta a_t}{{\,\mathrm{\textrm{e}}\,}}^{\frac{\theta }{2}\partial _{a_t}F\left( {1\over q},t\right) }\left( \frac{1}{1-t}\right) ^{\theta a_t}\,\\&\quad \mathcal {F}\left( \begin{matrix} a_{q} \\ a_{\infty } \end{matrix} \, b_2 \, \begin{matrix} a_1 \\ \, \end{matrix} \,b_1 \, \begin{matrix} a_0 \\ \, \end{matrix} \,a_{t\theta } \, \begin{matrix} a_{2,1} \\ a_t \end{matrix} ; \frac{1-t}{q-t}, \frac{t}{t-1}, \frac{-z+t}{t} \right) \\&= \left( {1-t}\right) ^{\frac{1}{2}}t^{\theta a_t}{{\,\mathrm{\textrm{e}}\,}}^{\frac{\theta }{2}\partial _{a_t}F\left( {1\over q},t\right) }\,\mathcal {F}\left( \begin{matrix} a_{q} \\ a_{\infty } \end{matrix} \, b_2 \, \begin{matrix} a_1 \\ \, \end{matrix} \,b_1 \, \begin{matrix} a_0 \\ \, \end{matrix} \,a_{t\theta } \, \begin{matrix} a_{2,1} \\ a_t \end{matrix} ; \frac{1-t}{q-t}, \frac{t}{t-1}, \frac{-z+t}{t} \right) ,\end{aligned}\end{aligned}$$

**Table 1:** Check of numerical values of $\mathcal {C}_{t,-}^{0,+}$ at the parameters (3.18)

Number of instantons	Real part	Imaginary part
0	− **14**2813.86214	− **39**2377.86142
1	− **1436**23.93208	− **3946**03.51028
2	− **143630**.89467	− **394622**.63983
3	− **143630**.**92**880	− **394622**.**7**3363
Wronskian	− 143630.92949	− 394622.73552

**Table 2:** Check of numerical values of $\mathcal {C}_{t,-}^{0,-}$ at the parameters (3.18)

Number of instantons	Real part	Imaginary part
0	**0**.**0**3884950457	**0**.**1**0673813655
1	**0**.**0404**7079339	**0**.**111**19259100
2	**0**.**040481**02795	**0**.**111220**71021
3	**0**.**0404810**7976	**0**.**11122085**255
Wronskian	0.04048108053	0.11122085466

**Table 3:** Check of numerical values of $\mathcal {C}_{t,-}^{0,+}$ at the parameters (3.20)

Number of instantons	Real part	Imaginary part
0	− **38**80.678616	**7**802.008331
1	− **3872**.550280	**7784**.393415
2	− **3872**.**9**14312	**7784**.**5**85712
3	− **3872**.**925**641	**7784**.**592**381
Wronskian	− 3872.925966	7784.592815

**Table 4:** Check of numerical values of $\mathcal {C}_{t,-}^{1,+}$ at the parameters (3.26)

Number of instantons	Real part	Imaginary part
0	− **16**9747.731	${\textbf {1.0}}79527249\times 10^6$
1	− **1683**85.977	${\textbf {1.070}}86704\times 10^6$
2	− **16837**0.680	${\textbf {1.07076}}9760\times 10^6$
3	− **168370**.**2**87	${\textbf {1.0707672}}60\times 10^6$
Wronskian	− 168370.3	$1.0707673\times 10^6$

**Table 5:** Check of numerical values of $\mathcal {C}_{t,-}^{1,-}$ at the parameters (3.26)

Number of instantons	Real part	Imaginary part
0	− **14**9860.161	− **9**53050.306
1	− **1486**45.277	− **945**324.135
2	− **148630**.420	− **94522**9.654
3	− **148630**.**1**02	− **945227**.**6**27
Wronskian	− 148630.1	945227.7

Thanks to crossing symmetry, we have

3.14 $$\begin{aligned}&{\mathrm e}^{\textrm{i}\pi (-\frac{1}{2}-\theta a_t)} \left( {1-t}\right) ^{\frac{1}{2}}\mathcal {F}\left( \begin{matrix} a_{\infty } \\ a_{q} \end{matrix} \, b_2 \, \begin{matrix} a_1 \\ \, \end{matrix} \,b_1 \, \begin{matrix} a_0 \\ \, \end{matrix} \,a_{t\theta } \, \begin{matrix} a_{2,1} \\ a_t \end{matrix} ; \frac{1-t}{q-t}, \frac{t}{(t-1)}, \frac{-z+t}{t} \right) \end{aligned}$$

3.15 $$\begin{aligned}&=\sum _{\theta '=\pm } \mathcal {M}_{\theta \theta '}(a_t,a_0;b_1) \mathcal {F}\left( \begin{matrix}a_q\\ a_{\infty }\end{matrix}\,b_2\,\begin{matrix}a_1\\ {}\end{matrix}\,b_1\,\begin{matrix} a_t\\ {}\end{matrix}\,a_{0\theta '}\,\begin{matrix}a_{2,1}\\ a_{0}\end{matrix};\frac{1}{q},t,\frac{z}{t}\right) , \end{aligned}$$

where the extra phase factor ${\mathrm e}^{\textrm{i}\pi (-\frac{1}{2}-\theta a_t)}$ is chosen to match the phases on both sides.^8^ The connection coefficients therefore read

3.16 $$\begin{aligned} \psi _{t,\theta }(z){=} {\mathrm e}^{\textrm{i}\pi \left( \frac{1}{2} + \theta a_t\right) } t^{\theta a_t} {\mathrm e}^{\frac{\theta }{2} \partial _{a_t} F\left( \frac{1}{q}, t\right) } \sum _{\theta '{=}\pm } \mathcal {M}_{\theta \theta '}(a_t, a_0; b_1) t^{{-}\theta ' a_0} {\mathrm e}^{-\frac{1}{2}\theta ' \partial _{a_0} F\left( \frac{1}{q}, t\right) } \psi _{0,\theta '}. \end{aligned}$$

Notice that the matrix $\mathcal {M}_{\theta \theta '}$ can be simply reabsorbed in the one-loop part of the NS function as in (A.6) and we have

3.17 $$\begin{aligned} \boxed {\begin{aligned} \psi _{t,\theta }(z)=&{\mathrm e}^{\textrm{i}\pi \left( \frac{1}{2} + \theta a_t\right) } t^{\theta a_t} {\mathrm e}^{\frac{\theta }{2} \partial _{a_t} F^\textrm{NS}\left( \frac{1}{q}, t,-\theta a_t\right) } \Gamma \left( 1+2 \theta a_t\right) \\&\sum _{\theta '=\pm } \Gamma \left( -2\theta ' a_0\right) t^{-\theta ' a_0}{{\,\mathrm{\textrm{e}}\,}}^{-{1\over 2}\theta ' \partial _{a_0} F^\textrm{NS} \left( \frac{1}{q}, t,-\theta a_t\right) } \psi _{0,\theta '}. \end{aligned}} \end{aligned}$$

The function $F^\textrm{NS}$ is defined in (A.6), and in (3.17) we have explicitly highlighted its dependence on $a_t$, as the dependences on the other $a_i$’s are fixed in the same manner as in (3.17), see the comment around (A.7). As an example, we choose the following values for the parameters

3.18 $$\begin{aligned} \begin{aligned} t&=\frac{1}{100},\quad q=200,\quad a_0=\frac{97}{70},\quad a_1=\frac{7}{141},\quad a_{\infty }=\frac{51}{40},\\ a_t&=\frac{10}{9},\quad a_{q}=\frac{4}{3},\quad \quad u_t=\frac{1}{50},\quad u_{q}=\frac{3}{8}, \end{aligned} \end{aligned}$$

and we check the values of the connection coefficients obtained as (3.17) against the ratios of Wronskians

3.19 $$\begin{aligned} \frac{W\left( \psi _{t,-}(z),\psi _{0,+}(z)\right) }{W\left( \psi _{0,-}(z),\psi _{0,+}(z)\right) }\quad {\text {and}}\quad \frac{W\left( \psi _{t,-}(z),\psi _{0,-}(z)\right) }{W\left( \psi _{0,+}(z),\psi _{0,-}(z)\right) }, \end{aligned}$$

where we compute 100 coefficients $c_j$ for each Frobenius solution (3.6).

We get the following results for the real and imaginary parts of the connection coefficients (Tables 1 and 2).

We finally show an example involving complex values of the parameters. Fixing

3.20 $$\begin{aligned} \begin{aligned} t&=\frac{3}{100}+\frac{1}{1000}\,\textrm{i},\quad q=700+4\,\textrm{i},\quad a_0=\frac{13}{11}+\frac{1}{10}\,\textrm{i},\quad \\ a_1&=\frac{2}{7}-\frac{1}{12}\,\textrm{i},\quad a_{\infty }=\frac{9}{8}+\frac{1}{9}\,\textrm{i},\\ a_t&=\frac{7}{6}-\frac{1}{11}\,\textrm{i},\quad a_{q}=\frac{11}{9},\quad \quad u_t=\frac{3}{40},\quad u_{q}=\frac{2}{5}, \end{aligned} \end{aligned}$$

at 3 instantons, replacing the phase factor of Eq. (3.16) with $\exp \left( i \pi \left( a_t-\frac{1}{2}\right) \right)$, we find the results in Table 3.

### Connection problem II

In this section, we study the connection formula for the solutions at z=t and z=1, in the regime $|t|\ll 1 \ll |q|$, as in Fig. 1. Let us take the singularities *t* and *q* lying on the positive real axis.^9^ For the local solutions around 1, we use the conformal blocks of the form3.21However, to our knowledge, combinatorial expressions for such conformal blocks have not been written down. Nevertheless, the connection coefficient can still be calculated as described in Sect. 2.2. As a normalization for these blocks, we propose3.22Let us work out the connection problem for the solutions around *t* and 1. Schematically, we follow the steps3.23We find

3.24 $$\begin{aligned} \begin{aligned} \psi _{t,\theta _1}(z)&= t^{\theta _1 a_t} {\mathrm e}^{\frac{\theta _1}{2}\partial _{a_t}F\left( {1\over q},t\right) } \sum _{\theta _2,\theta _3=\pm } \mathcal {M}_{\theta _1\theta _2}(a_t, b_1, a_0) \mathcal {M}_{(-\theta _2)\theta _3}(b_1, a_1, b_2) \\&\quad \times t^{\theta _2 b_1} {\mathrm e}^{-\frac{\theta _2}{2}\partial _{b_1}F\left( {1\over q},t\right) } {\mathrm e}^{\textrm{i}\pi \left( -\left( \frac{1}{2} + \theta _3 a_1\right) \right) } {\mathrm e}^{-\frac{\theta _3}{2}\partial _{a_1}F\left( {1\over q},t\right) }\psi _{1,\theta _3}(z) \end{aligned} \end{aligned}$$

This problem was also recently discussed in [65, App.B].

By using the full NS functions, we can write the following closed-form expression

3.25 $$\begin{aligned} \boxed {\begin{aligned} \psi _{t,\theta _1}(z)&= {2{{\,\mathrm{\textrm{e}}\,}}^{-\textrm{i}{\pi \over 2}} \pi t^{\theta _1 a_t} {\mathrm e}^{\frac{\theta _1}{2}\partial _{a_t}F^\textrm{NS}\left( {1\over q},t,-\theta _1 a_t\right) }\over \sin (2 \pi b_1)} \Gamma (2 a_t \theta _1 +1)\\  &\quad \sum _{\theta _3=\pm } {\mathrm e}^{\textrm{i}\pi \left( \theta _3 a_1\right) }\Gamma (-2 a_1 \theta _3 )\\&\quad \sinh \left( {1\over 2}\partial _{b_1}F^\textrm{NS}\left( {1\over q},t, -\theta _3 a_1, -\theta _1 a_t\right) \right) {\mathrm e}^{-\frac{\theta _3}{2}\partial _{a_1}F^\textrm{NS}\left( {1\over q},t, -\theta _3 a_1 \right) }\psi _{1,\theta _3}(z)~, \end{aligned}} \end{aligned}$$

where $F^\textrm{NS}$ is given in (A.6). As before, we use the convention that in the arguments of the $F^\textrm{NS}$ function, we explicitly write the dependence on the $a_i$ only when they have a different sign w.r.t. (A.6), see (A.7).

We check such expressions at parameters

3.26 $$\begin{aligned} t  &   =\frac{1}{100},\quad q=200,\quad a_0=\frac{97}{70},\quad a_1=\frac{7}{141},\quad a_{\infty }=\frac{51}{40},\nonumber \\ a_t  &   =\frac{10}{9},\quad a_{q}=\frac{4}{3},\quad \quad u_t=\frac{73}{50},\quad u_{q}=\frac{3}{8}\ . \end{aligned}$$

The comparison of numerical and instanton results for the connection coefficient of $\psi _{t,-}$ and $\psi _{1,\pm }$ is given in Tables 4 and 5.

### Connection problem III

In this section, we study the connection formula for the solutions at z=1 and z=q, in the regime $|t|\ll 1 \ll |q|$, as in Fig. 1. Let us take the singularities *t* and *q* lying on the positive real axis. For the local solutions around 1, we use a conformal block of the form (3.21).

Schematically, we use the following steps3.27We find

3.28 $$\begin{aligned} \begin{aligned}\psi _{1,\theta _3}(z)&= {\mathrm e}^{\frac{\theta _3}{2}\partial _{a_1}F\left( {1\over q},t\right) } \sum _{\theta _4,\theta _5=\pm } \mathcal {M}_{\theta _3\theta _4}(a_1, b_2, b_1) \mathcal {M}_{(-\theta _4)\theta _5}(b_2, a_q, a_\infty ) \\&\quad \times q^{-\theta _4 b_2} {\mathrm e}^{-\frac{\theta _4}{2}\partial _{b_2}F\left( {1\over q},t\right) } q^{-\theta _5 a_q} {\mathrm e}^{-\frac{\theta _5}{2}\partial _{a_q}F\left( {1\over q},t\right) } {\mathrm e}^{\textrm{i}\pi \left( -\left( \frac{1}{2} + \theta _5 a_q\right) \right) }\psi _{q,\theta _5}(z). \end{aligned}\end{aligned}$$

As before, we can write this by using the full NS function and we get

3.29 $$\begin{aligned} \boxed {\begin{aligned} \psi _{1,\theta _3}(z)&= {2 \pi {{\,\mathrm{\textrm{e}}\,}}^{-{\textrm{i}\pi \over 2} }{\mathrm e}^{\frac{\theta _3}{2}\partial _{a_1}F^\textrm{NS}\left( {1\over q},t,\theta _3 a_1\right) } \over \sin (2 \pi b_2)}\Gamma (1+2 \theta _3 a_1 )\sum _{\theta _5=\pm } \Gamma (-2\theta _5 a_q) {{\,\mathrm{\textrm{e}}\,}}^{- \textrm{i}\pi \theta _5 a_q}\\&\sinh \left( {1\over 2}\partial _{b_2}F^\textrm{NS}\left( {1\over q},t,\theta _3 a_1,\theta _5 a_q \right) \right) {{\,\mathrm{\textrm{e}}\,}}^{-\frac{\theta _5}{2}\partial _{a_q}F^\textrm{NS}\left( {1\over q},t, \theta _5 a_q\right) } q^{-\theta _5 a_q}\psi _{q,\theta _5}(z), \end{aligned}} \end{aligned}$$

where $F^\textrm{NS}$ is given in (A.6). In (3.29), in the argument of the $F^\textrm{NS}$ function, we explicitly wrote the dependence only for the mass parameters $a_i$ that are subject to sign changes, see conventions in (A.7). We check this identity at the same parameter (3.26). The comparison of numerical and instanton results for the connection coefficient of $\psi _{1,-}$ and $\psi _{q,\mp }$ is given in Tables 6 and 7.

**Table 6:** Check of numerical values of $\mathcal {C}_{1,-}^{q,-}$ at the parameters (3.26)

Number of instantons	Real part	Imaginary part
0	**8**03.328333	− 463.801830
1	**77**2.013681	− **44**5.722307
2	**773**.**9**98712	− **446**.868365
3	**773**.**95**7904	− **446**.**8**44804
Wronskian	773.96	− 446.85

**Table 7:** Check of numerical values of $\mathcal {C}_{1,-}^{q,+}$ at the parameters (3.26)

Number of instantons	Real part	Imaginary part
0	${\textbf {0.0006}}44596393$	0.0003721579010
1	${\textbf {0.00059}}7936507$	${\textbf {0.00034}}5218803$
2	${\textbf {0.000599}}876502$	${\textbf {0.0003463}}38860$
3	${\textbf {0.0005998}}22354$	${\textbf {0.0003463}}07598$
Wronskian	0.0005998	0.0003463

### Connection problem IV

In this section, we study another connection formula for the solutions at z=t and z=q, in the regime $|t|\ll 1 \ll |q|$, as in Fig. 1.

The Frobenius solutions around z=t are related to the semiclassical limit of the conformal blocks by

3.30 $$\begin{aligned} \begin{aligned}\psi _{t,\theta }(z)&=t^{\theta a_t}\exp \left( \frac{\theta }{2}\partial _{a_t}F\left( {1\over q},t\right) \right) (z-q)\left( \frac{1-t}{(t-q)(1-q)}\right) ^{1/2}\\&\,\mathcal {F}\left( \begin{matrix} a_{\infty } \\ a_{q} \end{matrix} \, b_2 \, \begin{matrix} a_1 \\ \, \end{matrix} \,b_1 \, \begin{matrix} a_0 \\ \, \end{matrix} \,a_{t\theta } \, \begin{matrix} a_{2,1} \\ a_t \end{matrix} ; \frac{1-t}{1-q}, \frac{t(1-q)}{q(1-t)}, \frac{q(z-t)}{t(z-q)} \right) , \end{aligned}\end{aligned}$$

where we applied the following coordinate transform to the conformal block (B.2).^10^

3.31 $$\begin{aligned} z\rightarrow \frac{(z-t)(1-q)}{(z-q)(1-t)}. \end{aligned}$$

The Frobenius solutions around z=q are related to the semiclassical limit of the conformal blocks by

3.32 $$\begin{aligned} \begin{aligned}\psi _{q,\theta }(z)&=q^{\theta a_q}\exp \left( \frac{\theta }{2}\partial _{a_q}F\left( {1\over q},t\right) \right) (z-t)\left( \frac{(1-q)}{(1-t)(q-t)}\right) ^{\frac{1}{2}} \\&\mathcal {F}\left( \begin{matrix} a_{0} \\ a_{t} \end{matrix} \, b_{1} \, \begin{matrix} a_{1} \\ \, \end{matrix} \,b_{2} \, \begin{matrix} a_{\infty } \\ \, \end{matrix} \,a_{q\theta } \, \begin{matrix} a_{2,1} \\ a_{q} \end{matrix} ; \frac{t(1-q)}{q(1-t)},\frac{1-t}{1-q}, \frac{z-q}{z-t}\right) , \end{aligned}\end{aligned}$$

where we applied the following coordinate transform to the conformal blocks (B.2)

3.33 $$\begin{aligned} z\rightarrow \frac{(z-q)(1-t)}{(z-t)(1-q)}. \end{aligned}$$

As discussed in (2.33), different phase factors must be considered depending on the arguments of *t* and *q*. Let us now work out a few examples in detail.

#### q<t<0

To find the connection coefficients, we go a step back to the conformal blocks before the semiclassical limit is taken. The conformal block around z=t is presented by:3.34Explicitly, the conformal block together with the Jacobian factors for (3.34) is:

3.35 $$\begin{aligned}&q^{-2\Delta _{0}}(z-q)^{-2\Delta _{2,1}}(1-t)^{\Delta _{q}-\Delta _{\infty }-\Delta _{2,1}-\Delta _0-\Delta _t-\Delta _1}(1-q)^{\Delta _{\infty }+\Delta _{2,1}+\Delta _0+\Delta _t-\Delta _1-\Delta _{q}}\nonumber \\&\quad \times (t-q)^{\Delta _{\infty }+\Delta _{2,1}+\Delta _0+\Delta _1-\Delta _t-\Delta _{q}}\mathfrak {F}\left( \begin{matrix} \alpha _{\infty } \\ \alpha _{q} \end{matrix} \, \beta _2 \, \begin{matrix} \alpha _1 \\ \, \end{matrix} \,\beta _1 \, \begin{matrix} \alpha _0 \\ \, \end{matrix} \,\alpha _{t\theta } \, \begin{matrix} \alpha _{2,1} \\ \alpha _t \end{matrix} ; \frac{1-t}{1-q},\frac{t}{q}\frac{1-q}{1-t},\frac{q(z-t)}{t(z-q)} \right) . \end{aligned}$$

Similarly, we study the conformal block around z=q3.36Explicitly, the conformal block together with the Jacobian factors for (3.36) is:

3.37 $$\begin{aligned} \begin{aligned}&t^{-2\Delta _0}(z-t)^{-2\Delta _{2,1}}(1-t)^{\Delta _{\infty }+\Delta _{2,1}+\Delta _0+\Delta _{q}-\Delta _t-\Delta _1}(1-q)^{\Delta _{t}-\Delta _{\infty }-\Delta _{2,1}-\Delta _0-\Delta _{q}-\Delta _1}\\&\quad \times (q-t)^{\Delta _{\infty }+\Delta _{2,1}+\Delta _0+\Delta _1-\Delta _t-\Delta _{q}}\mathfrak {F}\left( \begin{matrix} \alpha _{0} \\ \alpha _{t} \end{matrix} \, \beta _1 \, \begin{matrix} \alpha _1 \\ \, \end{matrix} \,\beta _2 \, \begin{matrix} \alpha _{\infty } \\ \, \end{matrix} \,\alpha _{q\,\theta } \, \begin{matrix} \alpha _{2,1} \\ \alpha _{q} \end{matrix} ; \frac{t}{q}\frac{1-q}{1-t},\frac{1-t}{1-q},\frac{z-q}{z-t} \right) . \end{aligned} \end{aligned}$$

In order to obtain the connection coefficients connecting (3.35) and (3.37), we follow the steps3.38In each step, we used (2.38) for moving $\alpha _{2,1}$ around. For example, the first step can be written as:3.39Putting the steps all together, the connection formula between the conformal blocks reads^11^

3.40 $$\begin{aligned} \begin{aligned}&q^{-2\Delta _{0}}(z-q)^{-2\Delta _{2,1}}(1-t)^{\Delta _{q}-\Delta _{\infty }-\Delta _{2,1}-\Delta _0-\Delta _t-\Delta _1}(1-q)^{\Delta _{\infty }+\Delta _{2,1}+\Delta _0+\Delta _t-\Delta _1-\Delta _{q}}\\&\qquad \times (t-q)^{\Delta _{\infty }+\Delta _{2,1}+\Delta _0+\Delta _1-\Delta _t-\Delta _{q}}\mathfrak {F}\left( \begin{matrix} \alpha _{\infty } \\ \alpha _{q} \end{matrix} \, \beta _2 \, \begin{matrix} \alpha _1 \\ \, \end{matrix} \,\beta _1 \, \begin{matrix} \alpha _0 \\ \, \end{matrix} \,\alpha _{t\theta _1} \, \begin{matrix} \alpha _{2,1} \\ \alpha _t \end{matrix} ; \frac{1-t}{1-q},\frac{t}{q}\frac{1-q}{1-t},\frac{q(z-t)}{t(z-q)} \right) \\&\quad =\sum _{\theta _2,\theta _3,\theta _4=\pm }\mathcal {M}_{\theta _1\theta _2}(b\alpha _t,b\beta _1;b\alpha _0)\mathcal {M}_{(-\theta _2)\theta _3}(b\beta _1,b\beta _2;b\alpha _1)\mathcal {M}_{(-\theta _3)\theta _4}(b\beta _2,b\alpha _{q};b\alpha _{\infty })\\&\qquad \times t^{-2\Delta _0}(z-t)^{-2\Delta _{2,1}}(1-t)^{\Delta _{\infty }+\Delta _{2,1}+\Delta _0+\Delta _{q}-\Delta _t-\Delta _1}(1-q)^{\Delta _{t}-\Delta _{\infty }-\Delta _{2,1}-\Delta _0-\Delta _{q}-\Delta _1}\\&\qquad \times (q-t)^{\Delta _{\infty }+\Delta _{2,1}+\Delta _0+\Delta _1-\Delta _t-\Delta _{q}}\mathfrak {F}\left( \begin{matrix} \alpha _{0} \\ \alpha _{t} \end{matrix} \, \beta _{1\,\theta _2} \, \begin{matrix} \alpha _1 \\ \, \end{matrix} \,\beta _{2\,\theta _3} \, \begin{matrix} \alpha _{\infty } \\ \, \end{matrix} \,\alpha _{q\,\theta _4} \, \begin{matrix} \alpha _{2,1} \\ \alpha _{q} \end{matrix} ; \frac{t}{q}\frac{1-q}{1-t},\frac{1-t}{1-q},\frac{z-q}{z-t} \right) , \end{aligned} \end{aligned}$$

which in the semiclassical limit becomes

3.41 $$\begin{aligned} \begin{aligned}&(z-q)(t-q)^{-\frac{1}{2}}(1-q)^{-\frac{1}{2}}(1-t)^{\frac{1}{2}}\mathcal {F}\left( \begin{matrix} a_{\infty } \\ a_{q} \end{matrix} \, b_2 \, \begin{matrix} a_1 \\ \, \end{matrix} \,b_1 \, \begin{matrix} a_0 \\ \, \end{matrix} \,a_{t\theta _1} \, \begin{matrix} a_{2,1} \\ a_t \end{matrix} ; \frac{1-t}{1-q}, \frac{t(1-q)}{q(1-t)}, \frac{(z-t)q}{(z-q)t} \right) \\&\quad \sum _{\theta _2,\theta _3,\theta _4=\pm }{\mathrm e}^{\textrm{i}\pi (-\frac{1}{2}-\theta _4 a_q)}\mathcal {M}_{\theta _1,\theta _2}(a_t,b_1;a_0)\mathcal {M}_{(-\theta _2),\theta _3}(b_1,b_2;a_1)\mathcal {M}_{(-\theta _3),\theta _4}(b_2,a_{q};a_{\infty })\\&\qquad \left( t\right) ^{\theta _2b_1}\left( q\right) ^{-\theta _3b_2}\exp \left( -\frac{\theta _2}{2}\partial _{b_1}F\left( {1\over q},t\right) -\frac{\theta _3}{2}\partial _{b_2}F\left( {1\over q},t\right) \right) \\&\qquad \times (z-t)(q-t)^{-\frac{1}{2}}(1-q)^{\frac{1}{2}}(1-t)^{-\frac{1}{2}} \mathcal {F}\left( \begin{matrix} a_{0} \\ a_{t} \end{matrix} \, b_{1} \, \begin{matrix} a_{1} \\ \, \end{matrix} \,b_{2} \, \begin{matrix} a_{\infty } \\ \, \end{matrix} \,a_{q\theta _4} \, \begin{matrix} a_{2,1} \\ a_{q} \end{matrix} ; \frac{t(1-q)}{q(1-t)},\frac{1-t}{1-q}, \frac{z-q}{z-t}\right) , \end{aligned} \end{aligned}$$

where we used (2.36). Written in terms of Frobenius solutions (up to phases),

3.42 $$\begin{aligned} \begin{aligned}&\psi _{t,\theta _1}(z)=t^{\theta _1 a_t}\exp \left( \frac{1}{2}\theta _1\partial _{a_t}F\left( {1\over q},t\right) \right) \\&\quad \times \sum _{\theta _2,\theta _3,\theta _4=\pm }\mathcal {M}_{\theta _1\theta _2}(a_t,b_1;a_0)\mathcal {M}_{(-\theta _2)\theta _3}(b_1,b_2;a_1)\mathcal {M}_{(-\theta _3)\theta _4}(b_2,a_{q};a_{\infty })\\&\quad \times t^{\theta _2b_1}q^{-\theta _3b_2}\exp \left( -\frac{\theta _2}{2}\partial _{b_1}F\left( {1\over q},t\right) -\frac{\theta _3}{2}\partial _{b_2}F\left( {1\over q},t\right) \right) \\&\quad \times q^{-\theta _4 a_q}\exp \left( -\frac{1}{2}\theta _4\partial _{a_q}F\left( {1\over q},t\right) \right) \psi _{q,\theta _4}(z). \end{aligned} \end{aligned}$$

In the case when q<t<0, by appropriately choosing the phase factor, (3.42) becomes

3.43 $$\begin{aligned} \begin{aligned}&\psi _{t,\theta _1}(z) = t^{\theta _1 a_t}\exp \left( \frac{1}{2}\theta _1\partial _{a_t}F\left( {1\over q},t\right) \right) \\&\quad \quad \times \sum _{\theta _2,\theta _3,\theta _4=\pm } {\mathrm e}^{\textrm{i}\pi \left( \frac{1}{2} - \theta _2 b_1 + \theta _3 b_2 + \theta _4 a_q\right) } \mathcal {M}_{\theta _1\theta _2}(a_t, b_1; a_0)\\&\quad \quad \mathcal {M}_{(-\theta _2)\theta _3}(b_1, b_2; a_1) \mathcal {M}_{(-\theta _3)\theta _4}(b_2, a_q; a_{\infty }) \\&\quad \quad \times t^{\theta _2 b_1} q^{-\theta _3 b_2} \exp \left( -\frac{\theta _2}{2}\partial _{b_1}F\left( {1\over q},t\right) -\frac{\theta _3}{2}\partial _{b_2}F\left( {1\over q},t\right) \right) q^{-\theta _4 a_q}\\&\quad \quad \exp \left( -\frac{1}{2}\theta _4\partial _{a_q}F\left( {1\over q},t\right) \right) \psi _{q,\theta _4}(z). \end{aligned} \end{aligned}$$

As before, we can write it in a closed form using the full NS function:

3.44 $$\begin{aligned} \boxed {\begin{aligned} \psi _{t,\theta _1}(z)&={ \pi {{\,\mathrm{\textrm{e}}\,}}^{\textrm{i}{\pi \over 2}}t^{\theta _1 a_t}{{\,\mathrm{\textrm{e}}\,}}^{\frac{1}{2}\theta _1\partial _{a_t}F^\textrm{NS}\left( {1\over q},t,-\theta _1 a_t\right) }\over \sin (2 \pi b_1) \sin (2 \pi b_2)}\Gamma (2 a_t\theta _1+1)\\&\times \sum _{\theta _2,\theta _3,\theta _4=\pm } {\mathrm e}^{\textrm{i}\pi \left( - \theta _2 b_1 + \theta _3 b_2 + \theta _4 a_q+{\theta _2-\theta _3\over 2}\right) } q^{-\theta _4 a_q} \Gamma (-2 \theta _4 a_q) \\&\quad \cos \left( \pi (a_1 + b_1 \theta _2 + b_2 \theta _3)\right) \\&\times {{\,\mathrm{\textrm{e}}\,}}^{-\frac{\theta _2}{2}\partial _{b_1}F^\textrm{NS}\left( {1\over q},t,-\theta _1 a_t, \theta _4 a_q\right) -\frac{\theta _3}{2}\partial _{b_2}F^\textrm{NS}\left( {1\over q},t,-\theta _1 a_t, \theta _4 a_q\right) -\frac{1}{2}\theta _4\partial _{a_q}F^\textrm{NS}\left( {1\over q},t,-\theta _1 a_t, \theta _4 a_q\right) } \psi _{q,\theta _4}(z), \end{aligned}} \end{aligned}$$

where $F^\textrm{NS}$ is defined in (A.6). As before, in the argument of the $F^\textrm{NS}$ function, we explicitly wrote the dependence only for the mass parameters $a_i$ that are subject to sign changes.

We check (3.43) at the parameters

3.45 $$\begin{aligned} \begin{aligned}&t=-\frac{1}{100},\quad q=-200,\quad a_0=\frac{97}{70},\quad a_1=\frac{7}{141},\quad a_{\infty }=\frac{51}{40},\\&a_t=\frac{10}{9},\quad \quad a_{q}=\frac{4}{3},\quad \quad u_t=\frac{73}{50},\quad u_{q}=\frac{3}{8}. \end{aligned} \end{aligned}$$

The comparison of numerical and instanton results for the connection coefficients of $\psi _{t,-}$ and $\psi _{q,\mp }$ for the values in (3.45) is given in Tables 8 and 9.

**Table 8:** Check of numerical values of $\mathcal {C}_{t,-}^{q,-}$ at the parameters (3.45)

Number of instantons	Real part	Imaginary part
0	${\textbf {4}}.82094126\times 10^8$	${\textbf {1.3}}24542726\times 10^9$
1	${\textbf {4.7}}1162611\times 10^8$	${\textbf {1.29}}4508635\times 10^9$
2	${\textbf {4.706}}14908\times 10^8$	${\textbf {1.2930}}03833\times 10^9$
3	${\textbf {4.70608}}452\times 10^8$	${\textbf {1.29298}}6095\times 10^9$
Wronskian	$4.70608\times 10^8$	$1.29299\times 10^9$

**Table 9:** Check of numerical values of $\mathcal {C}_{t,-}^{q,+}$ at the parameters (3.45)

Number of instantons	Real part	Imaginary part
0	− **36**7.655967	− **10**10.126467
1	− **372**.690209	− **1023**.957933
2	− **372**.**5**22917	− **1023**.**4**98302
3	− **372**.**532**782	− **1023**.**52**541
Wronskian	− 372.533	− 1023.53

#### q>t>0

In this example, the singularities *t* and *q* lie on the positive real axis. We separate the problem into two parts by studying the connection problem for the solutions around *t* and 1 or the solutions around 1 and *q*, respectively, and we use Sects. 3.3 and 3.4. All together, we write down the connection coefficients for the connection problem relating the Frobenius solutions around *t* and *q* along an upper semicircle contour avoiding the singularity at 1. We start with (3.24) and plug in (3.29):

3.46 $$\begin{aligned} \psi _{t,\theta _1}(z)&=t^{\theta _1 a_t}{\mathrm e}^{\frac{\theta _1}{2}\partial _{a_t}F\left( {1\over q},t\right) }\sum _{\theta _2,\theta _3=\pm }\mathcal {M}_{\theta _1\theta _2}(a_t,b_1,a_0)\mathcal {M}_{(-\theta _2)\theta _3}(b_1,a_1,b_2) \nonumber \\&\qquad \ \times \quad \ t^{\theta _2 b_1}{\mathrm e}^{-\frac{\theta _2}{2}\partial _{b_1}F\left( {1\over q},t\right) }{\mathrm e}^{\textrm{i}\pi \left( -\left( \frac{1}{2}+\theta _3 a_1\right) \right) }{\mathrm e}^{-\frac{\theta _3}{2}\partial _{a_1}F\left( {1\over q},t\right) }{\mathrm e}^{\frac{\theta _3}{2}\partial _{a_1}F\left( {1\over q},t\right) }\nonumber \\&\qquad \ \times \sum _{\theta _4,\theta _5=\pm }\mathcal {M}_{\theta _3\theta _4}(a_1,b_2,b_1)\mathcal {M}_{(-\theta _4)\theta _5}(b_2,a_q,a_\infty )\nonumber \\&\qquad \ \times q^{-\theta _4 b_2}{\mathrm e}^{-\frac{\theta _4}{2}\partial _{b_2}F\left( {1\over q},t\right) }q^{-\theta _5 a_q}{\mathrm e}^{-\frac{\theta _5}{2}\partial _{a_q}F\left( {1\over q},t\right) }{\mathrm e}^{\textrm{i}\pi \left( -(\frac{1}{2} +\theta _5 a_q)\right) }\psi _{q,\theta _5}(z)\nonumber \\&\quad =t^{\theta _1 a_t}{\mathrm e}^{\frac{\theta _1}{2}\partial _{a_t}F\left( {1\over q},t\right) }\sum _{\theta _2,\theta _3,\theta _4,\theta _5=\pm }\mathcal {M}_{\theta _1\theta _2}(a_t,b_1,a_0)\mathcal {M}_{(-\theta _2)\theta _3}(b_1,a_1,b_2)\nonumber \\&\qquad \ \times {\mathrm e}^{\textrm{i}\pi \left( -\left( \frac{1}{2}+\theta _3 a_1\right) \right) }\mathcal {M}_{\theta _3\theta _4}(a_1,b_2,b_1)\mathcal {M}_{(-\theta _4)\theta _5}(b_2,a_q,a_\infty ) t^{\theta _2 b_1}\nonumber \\&\qquad \ \times {\mathrm e}^{-\frac{\theta _2}{2}\partial _{b_1}F\left( {1\over q},t\right) } q^{-\theta _4 b_2}{\mathrm e}^{-\frac{\theta _4}{2}\partial _{b_2}F\left( {1\over q},t\right) }q^{-\theta _5 a_q}{\mathrm e}^{-\frac{\theta _5}{2}\partial _{a_q}F\left( {1\over q},t\right) }\nonumber \\&\qquad \ \times {\mathrm e}^{\textrm{i}\pi \left( -\left( \frac{1}{2} +\theta _5 a_q\right) \right) }\psi _{q,\theta _5}(z). \end{aligned}$$

Applying the identity

3.47 $$\begin{aligned}  &   \sum _{\theta _3=\pm }\mathcal {M}_{(-\theta _2)\theta _3}(b_1,a_1,b_2){\mathrm e}^{\textrm{i}\pi \left( -\left( \frac{1}{2}+\theta _3 a_1\right) \right) }\mathcal {M}_{\theta _3\theta _4}(a_1,b_2,b_1)\nonumber \\  &   \quad =\mathcal {M}_{(-\theta _2)\theta _4}(b_1,b_2,a_1){\mathrm e}^{\textrm{i}\pi (\theta _4 b_2-\theta _2 b_1)} \end{aligned}$$

we get

3.48 $$\begin{aligned} \begin{aligned}&\psi _{t,\theta _1}(z) =t^{\theta _1 a_t}{\mathrm e}^{\frac{\theta _1}{2}\partial _{a_t}F\left( {1\over q},t\right) }\\&\quad \ \times \sum _{\theta _2,\theta _4,\theta _5=\pm }\mathcal {M}_{\theta _1\theta _2}(a_t,b_1,a_0)\mathcal {M}_{(-\theta _2)\theta _4}(b_1,b_2,a_1){\mathrm e}^{\textrm{i}\pi (\theta _4 b_2-\theta _2 b_1)}\\&\quad \mathcal {M}_{(-\theta _4)\theta _5}(b_2,a_q,a_\infty ) t^{\theta _2 b_1}\\&\quad \ \times {\mathrm e}^{-\frac{\theta _2}{2}\partial _{b_1}F\left( {1\over q},t\right) } q^{-\theta _4 b_2}{\mathrm e}^{-\frac{\theta _4}{2}\partial _{b_2}F\left( {1\over q},t\right) }q^{-\theta _5 a_q}{\mathrm e}^{-\frac{\theta _5}{2}\partial _{a_q}F\left( {1\over q},t\right) }\\  &\quad {\mathrm e}^{\textrm{i}\pi \left( -\left( \frac{1}{2} +\theta _5 a_q\right) \right) }\psi _{q,\theta _5}(z). \end{aligned} \end{aligned}$$

Hence,

3.49 $$\begin{aligned} \begin{aligned}&\psi _{t,\theta _1}(z) = t^{\theta _1 a_t} {\mathrm e}^{\frac{\theta _1}{2}\partial _{a_t}F\left( {1\over q},t\right) } \\&\quad \times \sum _{\theta _2,\theta _3,\theta _4=\pm } \mathcal {M}_{\theta _1\theta _2}(a_t,b_1,a_0) \mathcal {M}_{(-\theta _2)\theta _3}(b_1,b_2,a_1)\\&\quad \mathcal {M}_{(-\theta _3)\theta _4}(b_2,a_q,a_\infty ) t^{\theta _2 b_1} {\mathrm e}^{-\frac{\theta _2}{2}\partial _{b_1}F\left( {1\over q},t\right) } \\&\quad \times q^{-\theta _3 b_2} {\mathrm e}^{-\frac{\theta _3}{2}\partial _{b_2}F\left( {1\over q},t\right) } q^{-\theta _4 a_q} {\mathrm e}^{-\frac{\theta _4}{2}\partial _{a_q}F\left( {1\over q},t\right) } {\mathrm e}^{\textrm{i}\pi \left( \theta _3 b_2 - \theta _2 b_1\right) } {\mathrm e}^{\textrm{i}\pi \left( -\left( \frac{1}{2} + \theta _4 a_q\right) \right) } \psi _{q,\theta _4}(z). \end{aligned} \end{aligned}$$

We check this at the parameters in (3.26). The comparison of numerical and instanton results for the connection coefficients of $\psi _{t,-}$ and $\psi _{q,\mp }$ is given in Tables 10 and 11.

**Table 10:** Check of numerical values of $\mathcal {C}_{t,-}^{q,-}$ at the parameters (3.26)

Number of instantons	Real part	Imaginary part
0	${\textbf {6}}.5011735\times 10^8$	${\textbf {1}}.24167927\times 10^9$ i
1	${\textbf {6.8}}270759\times 10^8$	${\textbf {1.26}}157640\times 10^9$ i
2	${\textbf {6.810}}4579\times 10^8$	${\textbf {1.260}}54896\times 10^9$ i
3	${\textbf {6.8107}}137\times 10^8$	${\textbf {1.260557}}70\times 10^9$ i
Wronskian	$6.8107\times 10^8$	$1.260558\times 10^9$ i

**Table 11:** Check of numerical values of $\mathcal {C}_{t,-}^{q,+}$ at the parameters (3.26)

Number of instantons	Real part	Imaginary part
0	− **5**86.254383	**9**09.002256 i
1	− **565**.545454	**90**4.228989 i
2	− **565**.**9**33719	**903**.463207 i
3	− **565**.**90**7003	**903**.**44**6140 i
Wronskian	− 565.908	903.447 i

## A few applications

We now discuss some applications of our results in the context of black-hole perturbation theory.

There are many problems in black-hole perturbation theory in which the perturbation equation has five regular singularities. These include (generic) massive scalar perturbations of Schwarzschild-(A)dS in four and seven dimensions, as well as of Kerr-(A)dS in four dimensions and of Reissner–Nordström-(A)dS in four dimensions. In the rotating and higher-dimensional cases, the black-hole solutions to Einstein equations are not unique as there can be multiple rotation axes and different regimes in which the singularity structure of the perturbation equation changes [66–71], see Appendix D for some examples. Among these, we find that the massive scalar perturbation has five regular singularities in the seven-dimensional case with a single rotation parameter, or in the hyperbolic membrane limit, and in the nine-dimensional case in the black brane and ultraspinning regime. The same singularity structure arises also when considering different types of perturbations. For example, this happens for the scalar-sector of gravitational perturbations in Schwarzschild-(A)dS in four dimensions as studied in [72], and for the angular equation satisfied by the quasinormal modes of the charged C-metric [73]. For charged black holes, perturbations of electromagnetic fields are non-trivially coupled to the vector modes of metric perturbations. In [74], it was shown that it is possible to reduce the system of perturbation equations to two decoupled ODEs by taking appropriate combinations of the gauge-invariant quantities. In five dimensions, such perturbation equation has the singularity structure of our interest, as we will analyze in Sect. 4.2. We refer to [75] for a recent review of the singularity structure of equations governing black-hole perturbation theory.

Once the Seiberg–Witten to gravity dictionary is established, several applications can be explored. Here we consider applications in the framework of the AdS/CFT correspondence, where a black hole in ${\text {AdS}}_{d+1}$ is dual to a *d*-dimensional CFT at finite temperature. In this setting, the ratio of connection coefficients in the wave equation directly computes the thermal two-point functions in the dual CFT [76–79]. By further assuming the Eigenstate Thermalization Hypothesis and focusing on the large-spin regime, we can use the thermal two-point function to extract both the anomalous dimensions and the three-point functions for a class of double-twist operators [80–85]. This was used in [86] where, in the example of a scalar perturbation, an explicit solution to the heavy-light-cone bootstrap in four dimensions was provided via the NS functions. See also [54, 87–92] for an analogous computation of the thermal two-point functions in different backgrounds or under other types of perturbations. In this section, we use the results of Sect. 3 to extend the analysis to include scalar perturbations in Schwarzschild-AdS_7_ black holes and perturbations describing electromagnetic fields coupled to vector modes of the metric in Reissner–Nordström-AdS_5_ black holes.

### Scalar perturbations Schwarzschild-AdS_7_ black hole

The Schwarzschild-AdS_7_ black-hole metric is given by

4.1 $$\begin{aligned} \textrm{d}s^2=-f(r)\textrm{d}t^2+f(r)^{-1}\textrm{d}r^2+r^2\textrm{d}\Omega ^2_5, \end{aligned}$$

with

4.2 $$\begin{aligned} f(r)=1-\frac{\mu }{r^4}-\frac{\Lambda }{15}r^2, \end{aligned}$$

where μ is related to the mass *M* of the black hole via^12^

4.3 $$\begin{aligned} M=\frac{5\,\pi ^2\,\mu }{16}. \end{aligned}$$

In what follows, we will normalize the AdS radius *L* to equal 1, or, equivalently, Λ=-15.

We further perturb the background (4.1) by a massive scalar field of mass ν. The corresponding Klein–Gordon equation can be encoded, after mode decomposition, in the following radial equation:

4.4 $$\begin{aligned} \left( \partial ^2_r + \frac{f'(r)}{f(r)} \partial _r +\frac{\omega ^2-V(r)}{f(r)^2}\right) \Phi (r)=0, \end{aligned}$$

where the potential is

4.5 $$\begin{aligned} V(r)= f(r)\left( \frac{\ell (\ell +4)+\frac{15}{4}}{r^2}+\frac{25\,\mu }{4 r^6}+\frac{35}{4}+\nu ^2\right) , \end{aligned}$$

see [93] and references therein.

Let us start by working out the SW theory to gravity dictionary. In the variable $y=r^2$, the function $y^2f(y)$ is a third-degree polynomial in *y* with one positive real root representing the BH horizon, $y_1$, and two real negative or complex conjugated roots (depending on the value of the black-hole mass), $y_2,y_3$. The two roots $y_2,y_3$ can be written in terms of $y_1$ as

4.6 $$\begin{aligned} y_2=\frac{-1-y_1-\sqrt{1-2y_1-3y_1^2}}{2},\quad y_3=\frac{-1-y_1+\sqrt{1-2y_1-3y_1^2}}{2}, \end{aligned}$$

and the full expression for $y_1$ is

4.7 $$\begin{aligned} y_1= {1\over 3}\left( {\textrm{D}} +\frac{1}{\textrm{D}}-1\right) , \quad \textrm{D}^3= \frac{27 \mu }{2}+\frac{3}{2} \sqrt{3} \sqrt{\mu (27 \mu -4)}-1~>0.\end{aligned}$$

For a generic value of the BH mass μ, the wave equation (4.4) has five regular singularities at $y=0, y_1, y_2, y_3,\infty$. However, for $\mu = \frac{4}{27}$, the singularities at $y_2$ and $y_3$ merge, resulting in a collision of two singular points. Consequently, the wave equation is transformed: it now has three regular singularities and one irregular singularity. It is not clear to us what is the meaning of this point.^13^ In the following, we will assume that μ does not lie in a neighborhood of this point. Specifically, we assume $\mu \notin [36/343, 3/8]$ so that 1/|q|<1 and we have the configuration of Fig. 1. It would be interesting to explore what implications this change in configurations might have on the black-hole side (if any), but we will not pursue that direction further here.

Under this assumption, we can introduce the new variable

4.8 $$\begin{aligned} z=\frac{y(y_3-y_2)}{y_3(y-y_2)}, \end{aligned}$$

and redefining the wave function as

4.9 $$\begin{aligned} \Phi (z)=\frac{z^{3/4}}{\sqrt{1-z}\, \root 4 \of {y_2+y_3 (z-1)}\, \sqrt{y_2 y_3 z-y_1 (y_2+y_3 (z-1))}}\,\psi (z), \end{aligned}$$

the differential equation satisfied by ψ(z) can be written in the form (3.1) with the following dictionary^14^:

4.10 $$\begin{aligned} \begin{aligned} t=&\,\frac{y_1 (y_3-y_2)}{y_3(y_1-y_2)},\quad q=\,1-\frac{y_2}{y_3},\quad a_0=\,0,\quad a_t=\,\frac{i\,y_1^{3/2}\omega }{2(y_1-y_2)(y_1-y_3)},\\ a_1=&\,\frac{i\,y_3^{3/2}\omega }{2(y_1-y_3)(y_2-y_3)},\quad a_{q}=\,\frac{\sqrt{9+\nu ^2}}{2},\quad a_{\infty }=\,\frac{i\,y_2^{3/2}\omega }{2(y_1-y_2)(y_2-y_3)},\\ u_t=&\,t\,\Bigg (\frac{\omega ^2 y_1^2 y_3 (y_1-3 y_3)}{4 y_2 (y_1-y_3)^3 (y_2-y_3)}+\frac{\nu ^2 y_1 y_3 (y_2-y_1)}{4 y_2 (y_1-y_3) (y_2-y_3)}+\\&\,-\frac{\ell (\ell +4) y_3 (y_1-y_2)}{4 y_2 (y_1-y_3) (y_2-y_3)}-\frac{y_3 (y_1-y_2) (3 y_3 (y_1-y_2)+y_1 (14 y_1+y_2+10))}{4 y_1 y_2 (y_1-y_3) (y_2-y_3)}\Bigg ),\\ u_{q}=&\,q\,\frac{y_3 \left( 4 \ell (\ell +4)-4 \omega ^2+4 \nu ^2 (y_1-y_2+y_3)+31 y_1-33 y_2+31 y_3+15\right) }{16 y_2 (y_2-y_3)}. \end{aligned} \end{aligned}$$

In the *z* variable, the black-hole horizon is located at z=t and the AdS boundary is at z=q. As boundary conditions for the QNMs, we impose the vanishing Dirichlet boundary condition at z=q, and the presence of only ingoing modes at the horizon z=t. In terms of the wave function ψ, this means

4.11 $$\begin{aligned} \begin{aligned}&\psi (z)=\psi _{t,-}(z)\sim (z-t)^{\frac{1}{2}-\frac{i\omega y_1^{3/2}}{2(y_1-y_2)(y_1-y_3)}}&{\text {for}}\ z\sim t,\\&\psi (z)=\psi _{q,+}(z)\sim (z-q)^{\frac{1}{2}+\frac{\sqrt{9+\nu ^2}}{2}}&{\text {for}}\ z\sim q. \end{aligned} \end{aligned}$$

which are exactly the Frobenius solutions.

The connection formula between the Frobenius solutions expanded around z=t and z=q is given in (3.43). According to the boundary conditions (4.11), the quantization condition is given by:

4.12 $$\begin{aligned} \begin{aligned}&\sum _{\theta _2,\theta _3=\pm }\mathcal {M}_{-\theta _2}(a_t,b_1;a_0)\mathcal {M}_{(-\theta _2)\theta _3}(b_1,b_2;a_1)\mathcal {M}_{(-\theta _3)-}(b_2,a_{q};a_{\infty })\\&\quad \times t^{\theta _2b_1}\,q^{-\theta _3b_2}\exp \left( -\frac{\theta _2}{2}\partial _{b_1}F\left( {1\over q},t\right) -\frac{\theta _3}{2}\partial _{b_2}F\left( {1\over q},t\right) \right) =0. \end{aligned} \end{aligned}$$

In the framework of the AdS/CFT correspondence [94–96], a massive scalar field of mass ν in an $\textrm{AdS}_7$ background is dual a scalar operator of dimension Δ with

4.13 $$\begin{aligned} \nu ^2 = \Delta (\Delta - 6). \end{aligned}$$

A notable example of AdS_7_/CFT_6_ duality is the correspondence between M-theory on ${\text {AdS}}_7 \times S^4$ and the six-dimensional $\mathcal {N}=(2,0)$ superconformal field theory. This theory lacks of a Lagrangian description and very little is known about it. From this perspective, an analytic treatment of the perturbation equation in AdS_7_ is particularly compelling, since, by following [86], it can be used to extract OPE data for a class of operators in the 6d $\mathcal {N} = (2,0)$ theory.

In this approach, a key role is played by the scalar thermal two-point function which, in the language of the AdS_7_ wave equation, is simply given by the ratio of the connection coefficient [76–78]. From (3.44), we get

4.14 $$\begin{aligned} \begin{aligned}G_R(\omega , \ell )&=-\left( \frac{y_2 (y_3-y_2)}{ y_3}\right) ^{2 a_q}{W\left[ \psi _{t,-}, \psi _{q,-}\right] \over W\left[ \psi _{t,-},\psi _{q,+}\right] }\\&=\left( -y_2\right) ^{2 a_q} {\Gamma (-2 a_q)\over \Gamma (2 a_q)}{\mathcal {G} (a_0,a_1,a_t,a_q,a_{\infty }, t,q)\over \mathcal {G}(a_0,a_1,a_t,-a_q,a_{\infty }, t,q)}~\Big |_{\text {(4.10)}}~, \end{aligned}\end{aligned}$$

where the overall factor is due to the change of variables from *r* to *z* and

4.15 $$\begin{aligned} \begin{aligned}&\mathcal {G} (a_0, a_1, a_t, a_q, a_{\infty }, t, q) = {{\,\mathrm{\textrm{e}}\,}}^{-\frac{1}{2} \partial _{a_q}F^\textrm{NS}(1/q, t)} \Bigg ( \sinh \left( \frac{1}{2} \partial _{b_1}F^\textrm{NS}\left( \frac{1}{q}, t\right) \right) \\&\quad \sinh \left( \frac{1}{2} \partial _{b_2}F^\textrm{NS}\left( \frac{1}{q}, t, -a_1\right) \right) \\&\quad - {{\,\mathrm{\textrm{e}}\,}}^{-2 \textrm{i}a_1 \pi } \sinh \left( \frac{1}{2} \partial _{b_1}F^\textrm{NS}\left( \frac{1}{q}, t, -a_1\right) \right) \sinh \left( \frac{1}{2} \partial _{b_2}F^\textrm{NS}\left( \frac{1}{q}, t\right) \right) \Bigg )~. \end{aligned} \end{aligned}$$

The QNM’s frequencies are then identified as the poles of the thermal two-point function. Inspired by recent developments in the light-cone heavy-light bootstrap program [80–85], we consider the small $\mu \over \ell ^2$ expansion. In this limit, we have

4.16 $$\begin{aligned} b_1^{(0,0)}=\ell \left( \frac{1}{2\sqrt{2}}+\mathcal {O}(\mu \ell ^{-1})\right) ,\quad b_2^{(0,0)}=\ell \left( \frac{1}{2}+\mathcal {O}(\mu \ell ^{-1})\right) , \end{aligned}$$

and since within our range of parameters

4.17 $$\begin{aligned} \begin{aligned} 0<|t|<1,\quad \quad 0<\frac{1}{|q|}<1, \end{aligned} \end{aligned}$$

it follows that in the large ℓ regime

4.18 $$\begin{aligned} \begin{aligned} t^{b_1}\rightarrow 0,\quad \quad q^{-b_2}\rightarrow 0\, \end{aligned} \end{aligned}$$

are both exponentially suppressed. Therefore, if we neglect non-perturbative effects at large ℓ, equation (4.12) simplifies, as the terms with $\theta _2 = 1$ and $\theta _3 = -1$ are non-perturbative in 1/ℓ. The quantization condition then reduces to:

4.19 $$\begin{aligned} \begin{aligned} \frac{\Gamma \left( 2b_1\right) \Gamma \left( 1-2a_t\right) \Gamma \left( 1+2b_1\right) \Gamma \left( 2b_2\right) \Gamma \left( 1+2b_2\right) \Gamma \left( 2a_{q}\right) }{\prod _{\sigma =\pm }\Gamma \left( \frac{1}{2}-a_t+b_1+\sigma \,a_0\right) \Gamma \left( \frac{1}{2}+b_1+b_2+\sigma \,a_1\right) \Gamma \left( \frac{1}{2}+b_2+a_{q}+\sigma \,a_{\infty }\right) }=0. \end{aligned} \end{aligned}$$

This condition translates into requiring the presence of poles in $\Gamma \left( \frac{1}{2}+b_2+a_{q}\right. \left. +\sigma \,a_{\infty }\right)$, the only ones with arguments whose leading order in μ depends on ω. Note that the different sign of $a_{\infty }$ results in a different sign in the real part of ω. Without loss of generality, we choose the quantization condition

4.20 $$\begin{aligned} \frac{1}{2}+b_2+a_{q}+a_{\infty }=-n,\quad n\in \mathbb {Z}_{\ge 0}. \end{aligned}$$

As a quick reminder, $b_2$ is actually a function of the parameters in (4.10), which can be obtained by inverting the Matone relation

4.21 $$\begin{aligned} u_{q}=-\frac{1}{4}+b_2^2-a_{\infty }^2+a_{q}^2+q\frac{\partial F\left( {1\over q},t\right) }{\partial q}. \end{aligned}$$

In the inversion process, we also need the instanton expansion of $b_1$, which is obtained by inverting the other Matone relation

4.22 $$\begin{aligned} u_{t}=-\frac{1}{4}- b_1^2 + a_0^2 + a_t^2+t\frac{\partial F\left( {1\over q},t\right) }{\partial t}. \end{aligned}$$

Hence, upon using (4.10), Eq. (4.20) becomes a quantization condition for the frequency ω.

Note that, from (4.10), we have the following behavior

4.23 $$\begin{aligned} t\sim -1+3\sqrt{\mu },\quad \quad q\sim -\frac{1}{\sqrt{\mu }}+\frac{3}{2}+\mathcal {O}\left( \mu \right) \quad \quad {\text {as}}\quad \mu \sim 0~. \end{aligned}$$

Since |*t*| is not parametrically small, it is not immediately clear whether the approach based on the NS functions is effective. Nevertheless, thanks to the triangular-like expansion in $b_2$ given in (3.4), it suffices for 1/|*q*| to be parametrically small, which is indeed the case.^15^ Therefore, we can use the NS function to invert the quantization condition (4.20) and extract the QNM frequencies.

Since the coefficients in the $b_2$ expansion (3.4) behave as

4.24 $$\begin{aligned} b_2^{(K,K)}\left( \frac{t}{q}\right) ^{K}\propto \mu ^{K/2}, \end{aligned}$$

we expect the solutions to (4.20) to be of the form

4.25 $$\begin{aligned} \omega = \sum _{k \ge 0} \omega _k \left( \frac{\mu }{\ell ^2} \right) ^{k/4}. \end{aligned}$$

However, upon applying the SW to gravity dictionary, we find that all non-integer powers vanish because of some non-trivial cancelation which only happens when we use the SW to gravity dictionary in (4.10). For the first few coefficients, we find

4.26 $$\begin{aligned} \omega _0=&\,\ell +2n+\Delta , \nonumber \\ \omega _{1}=&\, \omega _{2}=\omega _3=0\nonumber \\ \omega _4=&\,-\frac{\ell ^2}{2 (\ell +1) (\ell +2) (\ell +3)}\Bigl \{3 \Delta ^2 \left( \ell (4 n-1)+4 n^2+6 n-3\right) \nonumber \\&+\Delta ^3 (\ell +2 n+3)+\nonumber \\&+2 \Delta \{3 \ell n (5 n-7)+\ell +n [n (10 n+9)-35]+3\} \nonumber \\&+10 (n-2) (n-1) n (2 \ell +n+3)\Bigr \}\nonumber \\ =&\, -\frac{1}{2} (\Delta +2 n-2) \left( (\Delta -1) \Delta +10 n^2+10 (\Delta -2) n\right) +O\left( \left( \frac{1}{\ell }\right) ^1\right) ~. \end{aligned}$$

In the context the AdS/CFT correspondence, the QNM expansion (4.25) should match the anomalous dimensions of heavy-light double-twist operators in the dual six-dimensional CFTs [81–85]. In fact, we can explicitly verify that the result (4.26) is in agreement with the anomalous dimensions computed in [97], hence providing an additional test of this correspondence. Higher-order terms in (4.25) can be computed by going to higher order in the NS function (A.4). This should be made more systematic either by using generalizations of Zamolodchikov recursion [98, 99] or by extending the method of [64, App.A]. However, we do not explore this further here.

### Electromagnetic and Gravitational perturbations Reissner–Nordström-AdS_5_

We now turn to an application of the connection formula (3.29). To this end, we consider a Reissner–Nordström-AdS_5_ black hole (RN), whose metric is given by:

4.27 $$\begin{aligned} \textrm{d}s^2=-f(r)\textrm{d}t^2+f(r)^{-1}\textrm{d}r^2+r^2\textrm{d}\Omega ^2_3, \end{aligned}$$

with

4.28 $$\begin{aligned} f(r)=1+r^2-\frac{2M}{r^2}+\frac{Q^2}{r^4}, \end{aligned}$$

where *M* and *Q* are the mass and the charge of the black hole, and where the AdS radius was normalized to 1 (that is, the cosmological constant is set to Λ=-6). In the variable $y=r^2$, the function $y^2f(y)$ is a third-degree polynomial in *y* with three roots

4.29 $$\begin{aligned} y^2f(y)= (y-y_h)(y-y_i)(y+1+y_h+y_i)~.\end{aligned}$$

Above extremity, we have two positive real roots, the bigger one, $y_h$, representing the event horizon and the smaller one, $y_i$, representing the inner horizon, and one real negative root, $y_n$. The two roots $y_i,y_n$ can be written in terms of $y_h$ as

4.30 $$\begin{aligned} y_i=\frac{-1-y_h+\sqrt{1-2y_h+8 M-3 y_h^2}}{2},\quad y_n=\frac{-1-y_h-\sqrt{1-2y_h+8 M-3 y_h^2}}{2}. \end{aligned}$$

The BH mass and charge are then given as:

4.31 $$\begin{aligned} M=-\frac{y_h\,y_i+y_h\,y_n+y_i\,y_n}{2} , \qquad Q^2=-y_i\,y_h\,y_n.\end{aligned}$$

The extremal limit of the geometry is reached in the confluence $y_h\rightarrow y_i$, that happens for the value of the charge

4.32 $$\begin{aligned} Q_{\text {ext}}=\frac{1}{3} \sqrt{\frac{2}{3}} \sqrt{(6 M+1)^{3/2}-9 M-1}. \end{aligned}$$

Massive scalar perturbations in this background are described by a second-order differential equation with four regular singularities; hence, the analysis is done exactly as in [86]. Electromagnetic and gravitational perturbations, on the other hand, are more challenging due to their non-trivial coupling. In [74, 100], the authors showed that these perturbation equations can be transformed into a set of two decoupled equations by introducing master variables written as linear combinations of gauge-invariant variables. Without sources, the differential equations read [100, eqs. (4.25)–(4.29)], [74, eq. (4.37)]

4.33 $$\begin{aligned} f(r)\frac{\textrm{d} }{\textrm{d} r}\left( f(r) \frac{\textrm{d} \Phi _{\pm } (r)}{\textrm{d} r}\right) +\left( \omega ^2-V_{\pm }(r)\right) \Phi _{\pm } (r)=0, \end{aligned}$$

where the potential is

4.34 $$\begin{aligned} V_{\pm }(r)= \frac{f(r)}{r^2}\left( k_V^2+\frac{39 Q^2}{4 r^4}+\frac{\mu _{\pm }}{r^2}+\frac{3 r^2}{4}+\frac{7}{4}\right) , \end{aligned}$$

with $$k_V^2=\ell (\ell +2)-1$$ and

4.35 $$\begin{aligned} \mu _{\pm }=-\frac{11}{2}M\pm \left[ 64M^2+12(k_V^2-2)Q^2\right] ^{1/2}. \end{aligned}$$

When Q=0, $\Phi _+$ reduces to the electromagnetic perturbation while $\Phi _-$ to the standard vector-type gravitational perturbation in Schwarzshild AdS_5_ background, [74, eq. (4.40)].

For a generic value of the charge *Q*, the master equation (4.33) has five regular singularities at $0,y_i,y_h, y_n$ and ∞. For $Q=Q_\textrm{ext}$, $y_h\rightarrow y_i$ and the equation develops an irregular singularity at the horizon, in addition to the regular singularities at y=0, $y=y_n$, and y=∞. On the gauge theory side, this corresponds to the decoupling limit of the quiver theory.

Let us take the differential equation satisfied by $\Phi _+$ (for the $\Phi _-$ case the procedure is the same). Introducing the new variable

4.36 $$\begin{aligned} z=\frac{y}{y_h}\frac{y_h-y_n}{y-y_n} \end{aligned}$$

and redefining the wave function as

4.37 $$\begin{aligned} \Phi _{+}(z)=\frac{z^{3/4}}{\sqrt{1-z} \root 4 \of {y_h (z-1)+y_n} \sqrt{y_h (-y_i z+y_i+y_n z)-y_i y_n}}\,\psi (z), \end{aligned}$$

the differential equation satisfied by ψ(z) can be written in the form (3.1) with the following dictionary:

4.38 $$\begin{aligned} \begin{aligned}\small t=&\,\frac{y_h y_i-y_i y_n}{y_h (y_i-y_n)},\quad q=\,\frac{y_h-y_n}{y_h},\quad a_0=\,2,\quad a_{q}=\,\frac{1}{2},\\ a_t=&\,\frac{i\,\omega \,y_i^{3/2}}{2 (y_h-y_i) (y_n-y_i)},\quad a_1=\,\frac{i\,\omega \, y_h^{3/2}}{2 (y_h-y_i) (y_h-y_n)},\quad a_{\infty }=\,\frac{i\,\omega \,y_n^{3/2}}{2 (y_h-y_n) (y_i-y_n)},\\ u_t=&\,\frac{3 y_h-y_i}{4 (y_h-y_i)}-\frac{y_i \left[ y_i \left( 4 k_V^2+12 y_h-y_i+7\right) -22 M\right] +39 Q^2}{16 y_i y_n (y_h-y_i)}\\&-\frac{\sqrt{3 \left( k_V^2-2\right) Q^2+16 M^2}}{2 y_n (y_h-y_i)}+\frac{ \omega ^2 y_i^3 (y_i-3 y_h)}{4 y_n (y_h-y_i)^3 (y_i-y_n)},\\ u_{q}=&\,\frac{-4 k_V^2+4 \omega ^2-8}{16 y_n}. \end{aligned} \end{aligned}$$

The differential equation satisfied by $\Phi _-$ has almost the same dictionary, but with a different sign in front of the square root term in $u_t$:

4.39 $$\begin{aligned} \begin{aligned} u_t=&\,\frac{3 y_h-y_i}{4 (y_h-y_i)}-\frac{y_i \left[ y_i \left( 4 k_V^2+12 y_h-y_i+7\right) -22 M\right] +39 Q^2}{16 y_i y_n (y_h-y_i)}\\&+\frac{\sqrt{3 \left( k_V^2-2\right) Q^2+16 M^2}}{2 y_n (y_h-y_i)}+\frac{ \omega ^2 y_i^3 (y_i-3 y_h)}{4 y_n (y_h-y_i)^3 (y_i-y_n)}. \end{aligned} \end{aligned}$$

In the *z* variable, the black-hole horizon is located at z=1 and the AdS boundary is at z=q. Therefore, the relevant problem is the one in Sect. 3.4. As boundary conditions for the QNMs, we impose the vanishing Dirichlet boundary condition at z=q, and the presence of only ingoing modes at the horizon z=1. In terms of the original wave function $\Phi _+(r)$ and of the tortoise coordinate $r_*=\int \textrm{d}r/f(r)$, we require

4.40 $$\begin{aligned} \begin{aligned}&\Phi _+(r)\sim \exp (-i\,\omega \,r_*)&{\text {for}}\ r\sim r_h,\\&\Phi _+(r)\sim r^{-3/2}&{\text {for}}\ r\rightarrow \infty . \end{aligned} \end{aligned}$$

In terms of the wave function ψ, this translates to

4.41 $$\begin{aligned} \begin{aligned}&\psi (z)=\psi _{1,-}(z)\sim (z-1)^{\frac{1}{2}-a_1}&{\text {for}}\ z\sim 1,\\&\psi (z)=\psi _{q,+}(z)\sim (z-q)^1&{\text {for}}\ z\sim q~. \end{aligned} \end{aligned}$$

Note that $a_q = \frac{1}{2}$ and $a_0 = 2$ correspond to logarithmic points of the differential equation, and the connection coefficients computed in Sect. 3.4 are singular at this point. However, as discussed in [54, 101], the quantization condition and the connection coefficients can be extracted from the non-logarithmic case $a_i \notin \mathbb {Z}/2$ by analytic continuation, which is equivalent to retaining the regular part as $a_i \rightarrow \mathbb {Z}/2$. This is the approach we adopt here. Therefore, the quantization condition arises from the connection formula between the semiclassical conformal blocks expanded around z=1 and the ones around z=q and is given by the requirement that the coefficient in front of the non-decaying solution at infinity is zero:

4.42 $$\begin{aligned} \begin{aligned} \sum _{\theta =\pm }\mathcal {M}_{-\theta }(a_1,b_2;b_1)\mathcal {M}_{(-\theta )-}(b_2,a_{q};a_{\infty })q^{-\theta \,b_2}\exp \left( -\frac{\theta }{2}\partial _{b_2}F\left( {1\over q},t\right) \right) =0. \end{aligned} \end{aligned}$$

From the perspective of AdS/CFT, the wave equation associated with gravitational and electromagnetic perturbations encodes information about the two-point functions of (mixed) stress-energy tensor and conserved currents, respectively [76, 79]. As in the scalar case the relevant quantity from which we extract these two-point functions is the ratio of connection coefficients

4.43 $$\begin{aligned} \begin{aligned}G_R=&-\left( \frac{y_n (y_h-y_n)}{y_h}\right) ^{2a_q}{W\left[ \psi _{1,-},\psi _{q,-}\right] \over W\left[ \psi _{1,-},\psi _{q,+}\right] }.\end{aligned}\end{aligned}$$

By using the (3.29), this reads

4.44 $$\begin{aligned} \begin{aligned}G_R=&y_n^{2a_q} {\Gamma \left( -2 a_q\right) \over \Gamma \left( 2 a_q\right) }{\mathcal {G}(a_0,a_1,a_t,a_q,a_{\infty },t,q)\over \mathcal {G}(a_0,a_1,a_t,-a_q, a_{\infty },t,q)}~\Big |_{\text {(4.38)}}\end{aligned}\end{aligned}$$

where

4.45 $$\begin{aligned} \mathcal {G}(a_0,a_1,a_t,a_q,a_{\infty },t,q)  &   =\cosh \left( {1\over 2}\partial _{b_2}F^\textrm{NS}\left( {1\over q},t,- a_1, a_q \right) \right) \nonumber \\    &   \quad {{\,\mathrm{\textrm{e}}\,}}^{-\frac{1}{2}\partial _{a_q}F^\textrm{NS}\big ({1\over q},t, a_1, a_q\big )}. \end{aligned}$$

As previously mentioned, for the black-hole geometry under consideration, we set $a_q = \frac{1}{2}$, which corresponds to a logarithmic singularity of the differential equation. Consequently, the equation (4.44) requires regularization. Following the approach in [54, 101], this is achieved by extracting the finite part.

Similarly to the case of scalar perturbations, the expressions here also simplify significantly in the small $\frac{M}{\ell }$ expansion. It is convenient to introduce

4.46 $$\begin{aligned} \tilde{Q}= {Q\over M}~.\end{aligned}$$

Let us first consider the quantization condition (4.42). Note that

4.47 $$\begin{aligned} b_2^{(0,0)} = \ell \left( \frac{1}{2} + \mathcal {O}\left( \frac{M}{\ell }, \frac{\tilde{Q}}{\ell }\right) \right) . \end{aligned}$$

Using the correspondence provided in (4.38), we note that

4.48 $$\begin{aligned} \frac{1}{|q|} < 1. \end{aligned}$$

This implies that the term proportional to $q^{-b_2}$ in (4.42) is exponentially suppressed for large ℓ. This result holds both for $\Phi _-$ and $\Phi _+$. Therefore, by neglecting non-perturbative effects in ℓ, the quantization condition simplifies to

4.49 $$\begin{aligned} \frac{1}{2}+b_2+a_{q}\pm a_{\infty }=-n,\quad n\in \mathbb {Z}_{\ge 0}. \end{aligned}$$

where the different ± signs in (4.49) correspond to a sign change in the real part of the QNM frequencies. The two instanton parameter behave as

4.50 $$\begin{aligned} \begin{aligned}q^{-1}&=2M +\mathcal {O}(M^2, M \tilde{Q}^2 ),\\ t&= {1\over 4}\tilde{Q}^2 + \mathcal {O}(M\tilde{Q}^2,\tilde{Q}^4), \end{aligned}\end{aligned}$$

suggesting that the natural approach would be a double expansion in M and $\tilde{Q}$. However, due to the triangular structure of the Matone relation (3.4), an expansion at small $\tilde{Q}$ is unnecessary for the QNMs. As a consequence, we consider the following expansion for the frequencies:

4.51 $$\begin{aligned} \omega ^{\pm }(Q,M,\ell ,n)=\sum _{j\ge 0}\omega _{j}^{\pm }(\ell ,n,\tilde{Q})\,\left( {M\over \ell }\right) ^j, \end{aligned}$$

where ± refers to the $\Phi _{\pm }$ perturbation.

Taking the quantization condition in (4.49) with the minus sign in front of $a_{\infty }$, we find for the first few orders $\omega _{0}^{\pm }(\ell ,n,\tilde{Q})=3 + \ell + 2 n$ and

4.52 $$\begin{aligned}  &   \omega _1^{\pm }(\ell ,n, \tilde{Q})=\,\frac{-2 (n+1) }{(\ell +1) (\ell +2)}\nonumber \\  &   \quad \biggl [3 \ell ^2 (n+1)\mp (\ell +n+2) \sqrt{3 (\ell -1) (\ell +3) \tilde{Q}^2+16}+\ell (6 n+11)+5 (n+2)\biggr ]. \end{aligned}$$

More orders are given in Appendix C. When $\tilde{Q}=0$ the structure simplifies significantly, and, in particular, no square roots are involved. We find that $\omega _{j}^{\pm }(\ell ,n,0)$ are polynomials in *n* of degree 2*j* and meromorphic functions in ℓ with poles at integers ℓ for $-j-1\le \ell \le j-1$.^16^ This behavior is very similar to the one of scalar perturbations in Schwarzschild AdS_5_, see [85].

In the large ℓ limit, we find

4.53 $$\begin{aligned} \omega _j^{\pm }(\ell ,n,\tilde{Q})=p_j^{\pm }(n,\tilde{Q})+\mathcal {O}\left( {1\over \ell }\right) , \end{aligned}$$

where $p_j^\pm (n,\tilde{Q})$ is a polynomial of degree j+1 in *n* and of degree *j* in $\tilde{Q}$. In addition, for the leading term in (4.53), we have

4.54 $$\begin{aligned} p_j^+(n,0)=p_j^-(n,0)=p_j(n) \end{aligned}$$

where $p_j(n)$ is the same polynomial as for scalar QNMs frequencies if we set the dimension of the scalar operator to be Δ=3 in [86, 97, 102, 103].

Let us now consider the ratio of connection coefficients (4.44). Neglecting the non-perturbative contributions in ℓ we find

4.55 $$\begin{aligned} \begin{aligned} G_R&\simeq y_n^{2a_q}\exp \left( -\partial _{a_{q}}F\left( {1\over q},t\right) \right) \frac{\Gamma \left( -2a_{q}\right) \Gamma \left( \frac{1}{2}+b_2+a_{q}+a_{\infty }\right) \Gamma \left( \frac{1}{2}+b_2+a_q-a_{\infty }\right) }{\Gamma \left( 2a_{q}\right) \Gamma \left( \frac{1}{2}+b_2-a_{q}+a_{\infty }\right) \Gamma \left( \frac{1}{2}+b_2-a_q-a_{\infty }\right) }. \end{aligned} \end{aligned}$$

We look for the residue of (4.55) at the poles of the Γ-function in the numerator given by

4.56 $$\begin{aligned} \frac{1}{2}+b_2+a_q-a_{\infty }=-n, \end{aligned}$$

which correspond to the QNMs in (4.51). We find

4.57 $$\begin{aligned} \begin{aligned} \textrm{Res}\left( G_R,\omega _{\text {QNM}}^{\pm }\right) =&y_n\,\exp \left( -\partial _{a_{q}}F\left( {1\over q},t\right) \right) \,(n+1)\,(2a_{\infty }-n-1) \\&\left[ \frac{\textrm{d}\left( b_2-a_{\infty }\right) }{\textrm{d}\,\omega }\right] ^{-1}\bigg |_{\omega =\text {(4.51)}}. \end{aligned} \end{aligned}$$

For simplicity, let us consider the case Q=0. We have

4.58 $$\begin{aligned} \begin{aligned} \textrm{Res}\left( G_R,\omega ={\text {(4.51)}}\right)&=\sum _{j\ge 0}c_{j}^\pm (\ell ,n)M^j, \end{aligned} \end{aligned}$$

where for the first few orders

4.59 $$\begin{aligned} \begin{aligned}c_0^+(\ell ,n)&=c_0^-(\ell ,n)=2(n+1)\,(\ell +n+2), \\ c_1^+(\ell ,n)&=-\frac{2(n+1)}{\ell (\ell +1) (\ell +2)}\times \\&\left[ 9 \ell ^3 (n+1)+\ell ^2 \left( 12 n^2+51 n+41\right) +\ell \left( 24 n^2+72 n+52\right) \right. \\&\left. +2 \left( 2 n^2+7 n+6\right) \right] ,\\ c_1^-(\ell ,n)&=c_1^+(\ell ,n)-\frac{16 (n+1) (\ell +n+2) (\ell +4 n+5)}{\ell (\ell +1) (\ell +2)}. \end{aligned}\end{aligned}$$

More results are given in Appendix C. As expected, the leading terms in the large ℓ expansion have the form

4.60 $$\begin{aligned} c_i^{\pm }(\ell ,n)={p_i(n)\over \ell ^i}+\mathcal {O}\left( {1\over \ell ^{i+1}}\right) ,\end{aligned}$$

where $p_i(n)$ are polynomials in *n* of degree i+1 and they coincide with the results in the μ-expansion in [86] with Δ=3 taking into account the additional overall factor $\frac{\ell +1}{2(\Delta -1)(\Delta -2)}=\frac{\ell +1}{4}$ from formula (38) in [86] and the fact that μ=2M.

The quantities above should be related to the OPE data of double-twist operators involving the stress tensor and a heavy operator. However, we are not aware of any available results on the bootstrap side for the case of the black-hole geometry considered here. It would be interesting to take the black brane limit and make connections with, e.g., [104]. We leave this for future work.

## Spectral network

Starting with [22], there has been a growing number of applications of the NS functions in the context of black-hole perturbation theory. In these applications, the NS functions are typically computed using instanton expansions, relying on expressions such as those provided in Appendix A and Appendix B. However, certain questions in gravity require expansions around specific values of the instanton counting parameters, as discussed in Sects. 4.2 and 4.1. In these cases, the instanton counting representation of the NS function proves useful, provided we operate in a regime where the instanton counting parameters (e.g., *t* and 1/*q*) are parametrically small. However, this condition is not always satisfied. For instance, when studying massive scalar perturbations of an $\textrm{AdS}_4$ Schwarzschild black hole, the wave equation takes the form (3.1), with instanton counting parameters

5.1 $$\begin{aligned} t=\frac{R_h (R_--R_+)}{R_- (R_h-R_+)}~,\quad {1\over q}={1\over 1-\frac{R_+}{R_-}}~,\end{aligned}$$

where

5.2 $$\begin{aligned} R_{\pm }=\frac{-R_h\pm i\sqrt{4R_h^2+3}}{2}~,\end{aligned}$$

and $R_h$ is the BH horizon. Hence, $|t|\in \left[ 0,{8\over \sqrt{65}}\right]$ and ${1\over |q|}\in \left[ {1 \over 2}, \frac{\sqrt{5}}{4}\right]$. Since $\frac{1}{q}$ is not parametrically small, we cannot even exploit the triangular structure of the Matone relation (3.4), making the approach based on instanton counting inefficient to explore the regime of interest for the light-cone bootstrap. Likewise, when studying the hydrodynamic limit [78], one needs to take a scaling limit where the black-hole mass goes to infinity. In this case, the instanton counting parameters are usually fixed to specific values (e.g., q=1/2 or t=1/2). Although these values lie within the radius of convergence of the instanton counting expressions, they are not parametrically small, making it difficult to extract useful information.

Therefore, in such situations, it would be valuable to have an alternative method for computing the NS functions that does not rely on instanton counting. This could be done via the NLIE approach discussed in [2, 105], the Zamolodchikov-TBA formalism of [106–108] or via the conformal limit of the GMN TBA [16, 17, 41, 42, 109–112]. The advantage of these methods is that they do not require the instanton counting parameters to be small. However, at least the GMN approach, is more practical when applied to the so-called strong-coupling region of the moduli space [113–115], as this region is characterized by a finite set of BPS particles and therefore a finite set of TBA equations. To transition from the strong to the weak-coupling region, one must cross the so-called wall of marginal stability, where BPS particles can decay or appear. Once the weak-coupling region is reached, the spectrum changes and it includes an infinite number of BPS particles; hence, to compute the NS functions via the GMN approach, one would need an infinite towers of coupled TBA equations.

In the black-hole setting, the gauge theory parameters are often constrained by non-trivial relations. It is therefore natural to ask whether the spectral problems associated with black-hole perturbation theory lie within the weak-coupling or strong-coupling region. This depends on the value of the frequency ω as well as the other black-hole parameters. By setting ω to one of the quasinormal modes (QNMs), we provide examples below where we show whether the relevant region corresponds to the strong-coupling region or weak-coupling region.

To determine the relevant region in the moduli space, we use the spectral network approach [116], see [117] for a recent review on their relevance in the context of $\mathcal {N}=2$ theories. A spectral network $\mathcal {W}^\vartheta$ depends on a phase factor ϑ and a quadratic differential λ which is defined by the ODE data in (2.46), that is

5.3 $$\begin{aligned} \lambda ^2=-P(z)\textrm{d}z^2, \end{aligned}$$

where *P*(*z*) is defined in (2.46). There are three walls emanated from each branch point *b*, which is denoted by an orange cross. And each wall comes with a label *ij*, where i,j=1,2, i≠j denoting the two sheets of (5.3)

5.4 $$\begin{aligned} \lambda ^{(i)}=(-1)^i\sqrt{-P(z)}\textrm{d}z \end{aligned}$$

and is given by a trajectory of *z* satisfying

5.5 $$\begin{aligned} \arg \left( -\int _{b}^z \left( \lambda ^{(i)}-\lambda ^{(j)}\right) \right) =\vartheta . \end{aligned}$$

In this section, we will neglect this label for convenience.

Spectral networks can be used to detect crucial information about the system. For example, an important application of it is to count BPS states. Graphically, BPS states show up as degenerate walls. In the *SU*(2) theories, a hypermultiplet consists of 2 head to head single walls—usually referred to as a saddle. On the other hand, a vectormultiplet corresponds to a ring domain whose boundary are degenerate walls. If we let the phase ϑ vary from 0 to 2π and record all the degenerate walls, this gives us the BPS spectrum of the supersymmetric gauge theory. Actually, the counting of BPS states also gives an important piecewise invariant of the theory, known as the BPS index [41, 118, 119], and the BPS index can tell us whether the parameters of supersymmetric gauge theory are in the strong-coupling region or the weak-coupling region.

We plot the spectral networks for scalar perturbations of Schwarzschild black hole, corresponding to *SU*(2) $N_f=3$ theory, the extremal Kerr black hole, corresponding to *SU*(2) $N_f=2$ theory, as well as AdS_5_-black brane, corresponding to *SU*(2) $N_f=4$ theory.

**Fig. 2:**
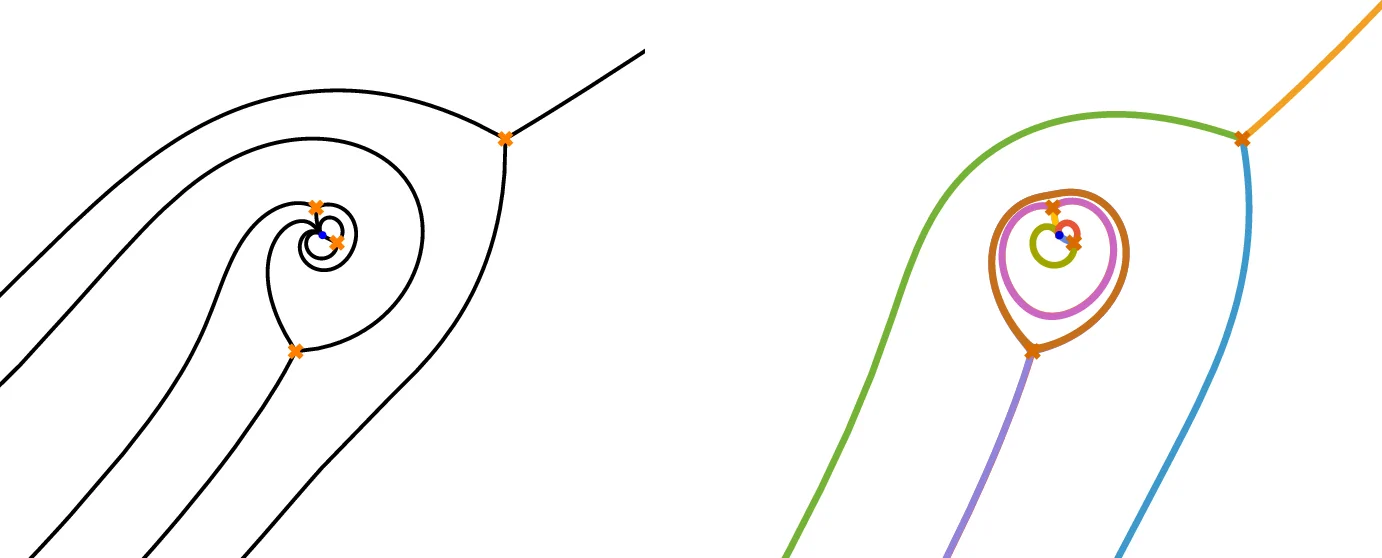
Spectral networks for extremal Kerr black hole with parameters in [22]. The BH quantum numbers are l=s=m=0. On the left we set ϑ=0 while on the right we set $\vartheta \approx 0.0872\pi$. In the second case, we see a ring domain in between the brown and pink degenerate walls

The spectral network for the extremal Kerr black is shown in Fig. 2. Varying the phase ϑ, we see a ring domain approximately at $\vartheta \approx 0.0872\pi$, which is shown in Fig. 2. This suggests that the black-hole parameters lie in the weak-coupling region.

**Fig. 3:**
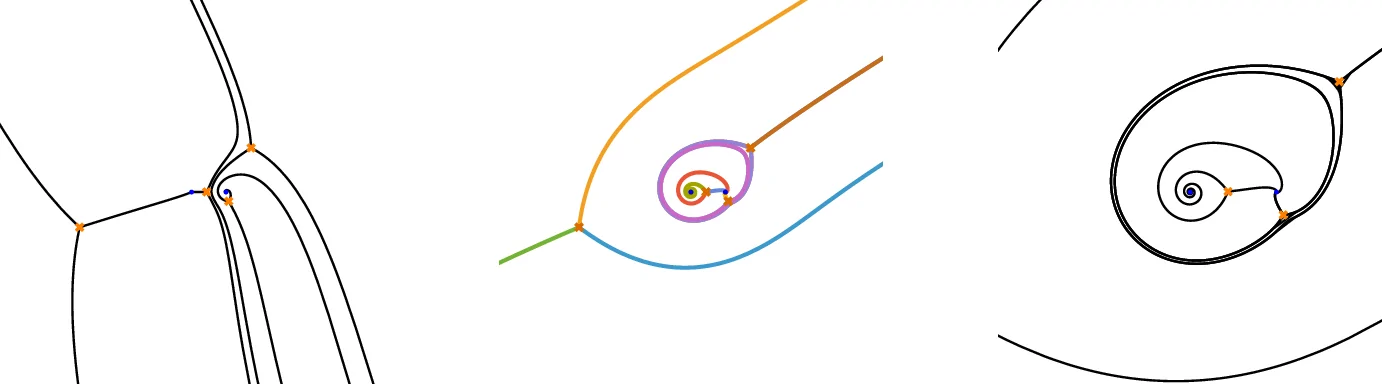
Spectral networks for Schwarzschild black hole corresponding to $N_f=3$ theory with parameters in [22]. The BH quantum number are n=0, l=s=1. In the left picture we set ϑ=0 while in the middle and right pictures we set $\vartheta \approx 0.5449\pi$. We see a ring domain in the middle picture in between the pink and purple degenerate walls. We zoom in further on the right picture

Likewise, the spectral network for the scalar perturbations of Schwarzschild black hole is shown in Fig. 3. Varying the phase ϑ, we see a ring domain approximately at $\vartheta \approx 0.5449\pi$. This indicates that the black-hole parameters lie in the weak-coupling region.

**Fig. 4:**
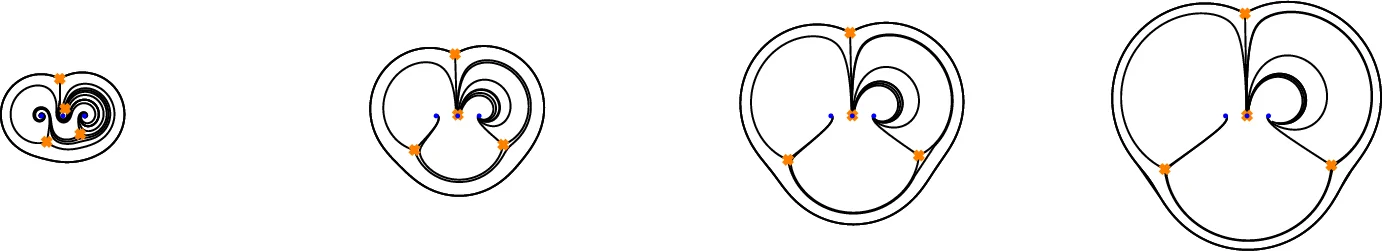
The first four quasinormal modes corresponding to $\Delta =2, \mathfrak {q}=0$ in the table 1 of [77]

We also consider the AdS_5_ black brane which is relevant for the hydrodynamic regime [77], whose wave equation is the one corresponding to the *SU*(2) $N_f=4$ theory. The spectral networks corresponding to the first 4 quasinormal modes at Δ=2 in the table 1 of [77] are shown in Fig. 4. Varying the phase ϑ, we see no ring domain. This indicates that such a spectral network belongs to a strong-coupling region. Moreover, we can see from Fig. 4 that the spectral networks also asymptotic to certain topology which is identified with an hypermultiplet. Similar results can be found using other Δ. Let us note that this system was also studied in a beautiful subsequent work [120]^17^ in the large-ω, large-q regime with fixed $\zeta ={\mathfrak q}/\omega$. The results presented there suggest the existence of two phase transitions: one occurring as q is turned on from zero, and another when ζ exceeds unity. Both transitions bear some resemblance to wall-crossing phenomena, and it would be interesting to understand this connection more precisely.

More generally, the appearance of string-coupling phases, open an intriguing avenue for exploring the interplay between GMN TBA and black-hole perturbation theory, a direction we leave for future investigations. Moreover, we expect that by varying the frequency and black-hole parameters, it may be possible to transition from the strong-coupling regime to the weak-coupling regime. It would be interesting to understand the implications of crossing the wall of marginal stability from the perspective of black holes. However, we leave this investigation for future work.

## Data Availability

The study did not involve any data.

## NS function without defect: the 5-point block

In this appendix, we define the NS partition function associated with the SU(2)×SU(2) linear quiver gauge theory discussed in Sect. 2.3, without any defects. The first step consists of defining the Nekrasov function.

For every Young diagram *Y*, we denote with $(Y_1\ge Y_2\ge \dots )$ the heights of its columns and with $(Y'_1\ge Y'_2,\dots )$ its rows’ lengths. For every box s=(i,j), we denote the arm length and the leg length of *s* with respect to the diagram *Y* as

A.1 $$\begin{aligned} A_Y(i, j) = Y_j - i, \quad L_Y(i, j) =Y'_i - j. \end{aligned}$$

We introduce the main contributions crucial for the definition of the instanton partition function with fundamental matter. We specify the notations that we use for the black-hole problems, where the five singular points are 0,1,∞,t,q, with |t|,1/|q|≪1.

We denote with $\vec {Y}=\left( Y_1, Y_2 \right)$ a pair of Young diagrams and with $\vec {b}_i=(b_{i,1},b_{i,2})$ the v.e.v. of the adjoint scalar in the vector multiplet for each gauge factor $SU(2)_i$, i=1,2, and with $\epsilon _1,\epsilon _2$ the parameters characterizing the Ω-background.

Let us introduce the main blocks involved in the definition of the instanton part of the NS function, that is the hypermultiplet, vector, and bifundamental contributions:

A.2 $$\begin{aligned} \begin{aligned}&E \left( b, Y_1, Y_2, (i,j) \right) = b - \epsilon _1 L_{Y_2} (i,j) + \epsilon _2 \left( A_{Y_1} (i,j) + 1 \right) \,,\\&z_{\text {hyp}} \left( \vec {b}, \vec {Y}, m \right) = \prod _{k= 1,2} \prod _{(i,j) \in Y_k} \left( b_k + m + \epsilon _1 \left( i - \frac{1}{2} \right) + \epsilon _2 \left( j - \frac{1}{2} \right) \right) \,, \\&z_{\text {vec}} \left( \vec {b}, \vec {Y} \right) = \prod _{k,l = 1,2} \prod _{(i,j) \in Y_k} E^{-1} \left( b_k - b_l, Y_k, Y_l, (i, j) \right) \\&\quad \prod _{(i',j') \in Y_l} \left( \epsilon _1+\epsilon _2 - E \left( b_l - b_k, Y_l, Y_k, (i', j') \right) \right) ^{-1}\,, \\&z_{\text {bif}}\left( \vec {b_1},\vec {Y},\vec {b_2},\vec {W};m_{\textrm{bif}} \right) \\&\quad =\prod _{k,l = 1,2} \prod _{(i,j) \in Y_k} \left( E \left( (b_1)_k - (b_2)_l, Y_k, W_l, (i, j) \right) -\left( \frac{\epsilon _1+\epsilon _2}{2}+m_{\textrm{bif}}\right) \right) \\&\quad \prod _{(i',j') \in W_l} \left( - E \left( (b_2)_l - (b_1)_k, W_l, Y_k, (i', j') \right) +\left( \frac{\epsilon _1+\epsilon _2}{2}-m_{\textrm{bif}}\right) \right) \,. \end{aligned} \end{aligned}$$

The Nekrasov function is then defined as:

A.3 $$\begin{aligned}&\mathfrak {F}\left( \begin{matrix}\alpha _q\\ \alpha _{\infty }\end{matrix}\,\beta _2\,\begin{matrix}\alpha _1\\ {}\end{matrix}\,\beta _1\,\begin{matrix}\alpha _t\\ \alpha _{0}\end{matrix};\frac{1}{q},t\right) \nonumber \\&\quad =q^{\Delta _{\infty }-\Delta _q-\Delta _{\beta _2}} t^{\Delta _{\beta _1} - \Delta _t - \Delta _{0}}\left( 1-\frac{1}{q}\right) ^{-2\left( \frac{Q}{2}+\alpha _q\right) \left( \frac{Q}{2}-\alpha _1\right) }(1-t)^{-2\left( \frac{Q}{2}+ \alpha _1\right) \left( \frac{Q}{2}-\alpha _t\right) } \nonumber \\&\qquad \left( 1-\frac{t}{q}\right) ^{-2\left( \frac{Q}{2}+\alpha _q\right) \left( \frac{Q}{2}+\alpha _t\right) } \nonumber \\&\qquad \times \sum _{\vec {Y},\vec {W}} \frac{t^{| \vec {Y} |}}{q^{|\vec {W}|}} z_{\text {vec}} \left( \vec {\beta _1}, \vec {Y} \right) z_{\text {vec}} \left( \vec {\beta _2}, \vec {W} \right) z_{\text {bif}} \left( \vec {\beta _1},\vec {Y},\vec {\beta _2},\vec {W};-\alpha _1 \right) \nonumber \\&\qquad \times \prod _{\sigma = \pm } z_{\text {hyp}} \left( \vec {\beta _1}, \vec {Y}, \alpha _t + \sigma a_0 \right) z_{\text {hyp}} \left( \vec {\beta _2}, \vec {W}, \alpha _{q} + \sigma \alpha _{\infty } \right) . \end{aligned}$$

Taking $\epsilon _1=1$ and $\vec {b_i}=(b_i,-b_i)$, i=1,2, the instanton part of the NS function is defined by taking $\epsilon _2\rightarrow 0$, that is

A.4 $$\begin{aligned} F\bigg ( \begin{matrix} a_{q} \\ a_{\infty } \end{matrix} \, b_2 \, \begin{matrix} a_1 \\ \, \end{matrix} \,b_1 \, \begin{matrix} a_t \\ a_0 \end{matrix} ; \frac{1}{q}, t \bigg )&=\lim _{\epsilon _2\rightarrow 0}\epsilon _2\log \Biggl [(1-t)^{-\frac{2 \left( a_1+\frac{1}{2}\right) \left( a_t+\frac{1}{2}\right) }{\epsilon _2}}\nonumber \\&\quad \left( 1-\frac{1}{q}\right) ^{-\frac{2 \left( \frac{1}{2}-a_1\right) \left( a_{q}+\frac{1}{2}\right) }{\epsilon _2}} \nonumber \\&\quad \left( 1-\frac{t}{q}\right) ^{-\frac{2 \left( a_t+\frac{1}{2}\right) \left( a_{q}+\frac{1}{2}\right) }{\epsilon _2}} \nonumber \\&\quad \times \sum _{\vec {Y},\vec {W}} \frac{t^{| \vec {Y} |}}{q^{|\vec {W}|}} z_{\text {vec}} \left( \vec {b_1}, \vec {Y} \right) z_{\text {vec}} \left( \vec {b_2}, \vec {W} \right) \prod _{\sigma = \pm } \nonumber \\&\quad z_{\text {hyp}} \left( \vec {b_1}, \vec {Y}, a_t + \sigma a_0 \right) z_{\text {hyp}} \left( \vec {b_2}, \vec {W}, a_{q} + \sigma a_{\infty } \right) \nonumber \\&\quad z_{\text {bif}} \left( \vec {b_1},\vec {Y},\vec {b_2},\vec {W};-a_1 \right) \Biggr ]. \end{aligned}$$

The first instanton contribution reads

A.5 $$\begin{aligned} \begin{aligned} F(1/q,t)=&\,\left[ t\,\frac{\left( 4a_0^2-4a_t^2-4b_1^2+1\right) \left( 4a_1^2+4b_1^2-4b_2^2-1\right) }{32b_1^2-8}\right. \\&\left. +\frac{1}{q}\,\frac{\left( 4a_1^2-4b_1^2+4b_2^2-1\right) \left( 4a_{\infty }^2-4a_{q}^2-4b_2^2+1\right) }{32b_2^2-8}\right] \\&+\mathcal {O}(t^2, q^{-2},t/q). \end{aligned} \end{aligned}$$

The full prepotential includes a classical and a 1-loop contribution in addition to the instanton part

A.6 $$\begin{aligned} F^{\text {NS}}(1/q,t)=&\,F(1/q,t)-b_1^2\,\log (t)-b_2^2\,\log (1/q)+\sum _{\theta =\pm }\psi ^{(-2)}(1+2\theta \,b_1) \nonumber \\&+\sum _{\theta =\pm }\psi ^{(-2)}(1+2\theta \,b_2) \nonumber \\&-\sum _{\theta _1,\theta _2=\pm }\psi ^{(-2)}\left( \frac{1}{2}+a_1+\theta _1\,b_1+\theta _2\,b_2\right) \nonumber \\&-\sum _{\theta ,\sigma =\pm }\psi ^{(-2)}\left( \frac{1}{2}+\theta \,b_1-a_t+\sigma \,a_0\right) \nonumber \\&-\sum _{\theta ,\sigma =\pm }\psi ^{(-2)}\left( \frac{1}{2}+\theta \,b_2-a_q+\sigma \,a_{\infty }\right) . \end{aligned}$$

This can be obtained from [21, App. B] and by using the NS limit of the $\Gamma _2$ functions, which can be found for instance in [12, (A.30)-(A.31)] . In (A.4) and (A.6), we have omitted the dependence on the $a_i$ parameters for the sake of simplicity in notation. However, in the main text, we occasionally use different signs for these parameters. This does not affect (A.4) but it does affect (A.6). Hence, in such cases, we explicitly indicate the dependence. For example, we note

A.7 $$\begin{aligned} F^\textrm{NS}\left( {1\over q},t, \theta a_k\right) ={\text {(A.6)}}\Big |_{a_k\rightarrow \theta a_k}, \quad \theta =\pm 1~. \end{aligned}$$

## NS function with defect: the degenerate 6-point block

The Nekrasov function in the presence of a canonical surface defect is defined by:

B.1 $$\begin{aligned}&\mathfrak {F}\left( \begin{matrix}\alpha _q\\ \alpha _{\infty }\end{matrix}\,\beta _2\,\begin{matrix}\alpha _1\\ {}\end{matrix}\,\beta _1\,\begin{matrix}\alpha _t\\ {}\end{matrix}\,\alpha _{0\theta }\,\begin{matrix}\alpha _{2,1}\\ \alpha _{0}\end{matrix};\frac{1}{q},t,\frac{z}{t}\right) =q^{\Delta _{\infty }-\Delta _q-\Delta _{\beta _2}} t^{\Delta _{\beta _1} - \Delta _t - \Delta _{0\theta }} z^{\frac{bQ}{2}+\theta b \alpha _0}\nonumber \\&\quad \left( 1-\frac{1}{q}\right) ^{-2\left( \frac{Q}{2}+\alpha _q\right) \left( \frac{Q}{2}-\alpha _1\right) }\nonumber \\&\quad \times (1-t)^{-2\left( \frac{Q}{2}+ \alpha _1\right) \left( \frac{Q}{2}-\alpha _t\right) }\left( 1-\frac{z}{t}\right) ^{-2(\frac{Q}{2}+ \alpha _t)\left( \frac{Q}{2}+\alpha _{2,1}\right) } \left( 1-\frac{t}{q}\right) ^{-2\left( \frac{Q}{2}+\alpha _q\right) \left( \frac{Q}{2}-\alpha _t\right) }\nonumber \\&\quad (1-z)^{-2\left( \frac{Q}{2}+ \alpha _1\right) \left( \frac{Q}{2}+\alpha _{2,1}\right) }\nonumber \\&\quad \times \left( 1-\frac{z}{q}\right) ^{-2\left( \frac{Q}{2}+\alpha _q\right) \left( \frac{Q}{2}+\alpha _{2,1}\right) }\sum _{\vec {Y}_1,\vec {Y}_2,\vec {Y}_3} \left( \frac{1}{q}\right) ^{| \vec {Y}_1 |}t^{| \vec {Y}_2 |} \left( \frac{z}{t}\right) ^{|\vec {Y}_3|} \nonumber \\&\quad z_{\text {vec}} \left( \vec {\beta }_2, \vec {Y}_1 \right) z_{\text {vec}} \left( \vec {\beta }_1, \vec {Y}_2 \right) z_{\text {vec}} \left( \vec {\alpha _{0\theta }}, \vec {Y}_3 \right) \nonumber \\&\quad \times z_{\text {bifund}} \left( \vec {\beta }_1,\vec {Y}_2,\vec {\beta }_2,\vec {Y}_1;-\alpha _1 \right) z_{\text {bifund}} \left( \vec {\beta }_1,\vec {Y}_2,\vec {\alpha _{0\theta }},\vec {Y}_3;\alpha _t \right) \nonumber \\&\quad \prod _{\sigma = \pm } z_{\text {hyp}} \left( \vec {\beta }_2, \vec {Y}_1, \alpha _q + \sigma \alpha _\infty \right) z_{\text {hyp}} \left( \vec {\alpha _{0\theta }}, \vec {Y}_3, \alpha _{2,1} + \sigma \alpha _0 \right) . \end{aligned}$$

The NS function in the presence of a canonical surface defect is then defined as:

B.2 $$\begin{aligned} \begin{aligned}&\mathcal {F}\left( \begin{matrix}a_q\\ a_{\infty }\end{matrix}\,b_2\,\begin{matrix}a_1\\ {}\end{matrix}\,b_1\,\begin{matrix} a_t\\ {}\end{matrix}\,a_{0\theta }\,\begin{matrix}a_{2,1}\\ a_{0}\end{matrix};\frac{1}{q},t,\frac{z}{t}\right) =\frac{\mathfrak {F}\left( \begin{matrix}\alpha _q\\ \alpha _{\infty }\end{matrix}\,\beta _2\,\begin{matrix}\alpha _1\\ {}\end{matrix}\,\beta _1\,\begin{matrix}\alpha _t\\ {}\end{matrix}\,\alpha _{0\theta }\,\begin{matrix}\alpha _{2,1}\\ \alpha _{0}\end{matrix};\frac{1}{q},t,\frac{z}{t}\right) }{\mathfrak {F}\left( \begin{matrix}\alpha _q\\ \alpha _{\infty }\end{matrix}\,\beta _2\,\begin{matrix}\alpha _1\\ {}\end{matrix}\,\beta _1\,\begin{matrix}\alpha _t\\ \alpha _{0}\end{matrix};\frac{1}{q},t\right) }\\&=t^{-a_{0\theta } }{\mathrm e}^{-\frac{1}{2}\theta \partial _{a_0}F\left( \frac{1}{q} , t\right) }(1-z)^{\frac{1}{2}+a_1}\left( 1-\frac{z}{q}\right) ^{\frac{1}{2} +a_q}\\  &\quad \left( 1-\frac{z}{t}\right) ^{\frac{1}{2} -a_t}z^{\frac{1}{2}+a_0 \theta }\left( 1+\mathcal O\left( \frac{z}{t}\right) \right) \\&= t^{-a_{0\theta } }{\mathrm e}^{-\frac{1}{2}\theta \partial _{a_0}F\left( \frac{1}{q} , t\right) } \psi _{0,\theta }(z), \end{aligned} \end{aligned}$$

where $F\left( \frac{1}{q}, t\right)$ is the shortcut for the NS free energy $$F\bigg ( \begin{matrix} a_{q} \\ a_{\infty } \end{matrix} \, b_2 \, \begin{matrix} a_1 \\ \, \end{matrix} \,b_1 \, \begin{matrix} a_t \\ a_0 \end{matrix} ; \frac{1}{q}, t \bigg )$$ in (A.4).

Note that $t^{-a_{0\theta } }{\mathrm e}^{-\frac{1}{2}\theta \partial _{a_0}F\left( \frac{1}{q}, t\right) }$ in (B.2) can be thought of as

B.3 $$\begin{aligned} t^{-a_{0\theta } }{\mathrm e}^{-\frac{1}{2}\theta \partial _{a_0}F\left( \frac{1}{q} , t\right) }={\mathrm e}^{-\frac{1}{2}\theta \partial _{a_0}\lim \limits _{b\rightarrow 0}b^2\log \mathfrak {F}\left( \begin{matrix}\alpha _q\\ \alpha _{\infty }\end{matrix}\,\beta _2\,\begin{matrix}\alpha _1\\ {}\end{matrix}\,\beta _1\,\begin{matrix}\alpha _t\\ \alpha _{0}\end{matrix};\frac{1}{q},t\right) }. \end{aligned}$$

## Frequencies and residue expansions

Some results for generic charge $\tilde{Q}$.

C.1 $$\begin{aligned} \omega _2^+(\ell ,n,\tilde{Q})&=\,\frac{(n+1)}{(\ell -1) \ell (\ell +1)^3 (\ell +2)^3 (\ell +3)} \biggl \{\ell ^9 \left[ 2 n (n+2) \left( 5 \tilde{Q}^2-17\right) +9 \tilde{Q}^2-39\right] \nonumber \\&\quad +\ell ^8 \biggl [12 (n+1) \sqrt{3 (\ell -1) (\ell +3) \tilde{Q}^2+16}+5 n^3 \left( \tilde{Q}^2-7\right) +3 n^2 \left( 35 \tilde{Q}^2-137\right) +n \left( 214 \tilde{Q}^2-802\right) \nonumber \\&\quad +105 \tilde{Q}^2-471\biggr ]+2 \ell ^7 \biggl \{\left( 18 n^2+84 n+74\right) \sqrt{3 (\ell -1) (\ell +3) \tilde{Q}^2+16}+20 n^3 \left( \tilde{Q}^2-7\right) \nonumber \\&\quad +3 n^2 \left( 73 \tilde{Q}^2-341\right) +2 n \left( 227 \tilde{Q}^2-983\right) +5 \left( 51 \tilde{Q}^2-239\right) \biggr \}+\ell ^6 \biggl \{n^3 \left( 113 \tilde{Q}^2-905\right) \nonumber \\&\quad +\left( 30 n^3+342 n^2+984 n+784\right) \sqrt{3 (\ell -1) (\ell +3) \tilde{Q}^2+16}+15 n^2 \left( 59 \tilde{Q}^2-363\right) \nonumber \\&\quad +2 n \left( 1025 \tilde{Q}^2-5291\right) +1446 \tilde{Q}^2-6770\biggr \}+\ell ^5 \biggl \{2 n^3 \left( 59 \tilde{Q}^2-755\right) +4 n^2 \left( 273 \tilde{Q}^2-2179\right) \nonumber \\&\quad +4 \left( 45 n^3+335 n^2+784 n+576\right) \sqrt{3 (\ell -1) (\ell +3) \tilde{Q}^2+16}+8 n \nonumber \\  &\quad \quad \left( 427 \tilde{Q}^2-2228\right) +3057 \tilde{Q}^2-12135\biggr \} \nonumber \\&\quad +\ell ^4 \biggl \{4 \left( 105 n^3+685 n^2+1437 n+987\right) \sqrt{3 (\ell -1) (\ell +3) \tilde{Q}^2+16}+n^3 \left( 179 \tilde{Q}^2-1457\right) \nonumber \\&\quad +n^2 \left( 2127 \tilde{Q}^2-9425\right) +n \left( 6274 \tilde{Q}^2-20518\right) +15 \left( 359 \tilde{Q}^2-969\right) \biggr \}+2 \ell ^3 \biggl \{26 n^3 \left( 13 \tilde{Q}^2-19\right) \nonumber \\&\quad +2 \left( 120 n^3+705 n^2+1366 n+899\right) \sqrt{3 (\ell -1) (\ell +3) \tilde{Q}^2+16}+n^2 \left( 2227 \tilde{Q}^2-3355\right) \nonumber \\&\quad +n \left( 4686 \tilde{Q}^2-7246\right) +3300 \tilde{Q}^2-5174\biggr \}+\ell ^2 (n+2) \biggl [n^2 \left( 723 \tilde{Q}^2+97\right) +3 n \left( 723 \tilde{Q}^2+97\right) \nonumber \\&\quad +\left( 118 n^2+354 n+468\right) \sqrt{3 (\ell -1) (\ell +3) \tilde{Q}^2+16}+1956 \tilde{Q}^2-504\biggr ]-2\ell (n+2) \biggl [-4 \left( 9 \tilde{Q}^2+266\right) \nonumber \\&\quad +\left( 122 n^2+366 n+220\right) \sqrt{3 (\ell -1) (\ell +3) \tilde{Q}^2+16}+\left( n^2+3 n\right) \left( 9 \tilde{Q}^2-565\right) \biggr ] \nonumber \\&\quad -12 (n+1) (n+2)^2 \left( 10 \sqrt{3 (\ell -1) (\ell +3) \tilde{Q}^2+16}+9 \tilde{Q}^2-41\right) \biggr \}, \end{aligned}$$

C.2 $$\begin{aligned} \omega _2^-(\ell ,n,\tilde{Q})&=\,-\frac{n+1}{(\ell -1) \ell (\ell +1)^3 (\ell +2)^3 (\ell +3)}\biggl \{\ell ^9 \biggl [n^2 \left( 34-10 \tilde{Q}^2\right) +n \left( 68-20 \tilde{Q}^2\right) -9 \tilde{Q}^2+39\biggr ]\nonumber \\&\quad +\ell ^8 \biggl [12 (n+1) \sqrt{3 (\ell -1) (\ell +3) \tilde{Q}^2+16}-5 n^3 \left( \tilde{Q}^2-7\right) +n^2 \left( 411-105 \tilde{Q}^2\right) \nonumber \\&\quad +n \left( 802-214 \tilde{Q}^2\right) -105 \tilde{Q}^2+471\biggr ]+\ell ^7 \biggl [4 \left( 9 n^2+42 n+37\right) \sqrt{3 (\ell -1) (\ell +3) \tilde{Q}^2+16}\nonumber \\&\quad -40 n^3 \left( \tilde{Q}^2-7\right) -6 n^2 \left( 73 \tilde{Q}^2-341\right) +n \left( 3932-908 \tilde{Q}^2\right) -510 \tilde{Q}^2+2390\biggr ]\nonumber \\&\quad +\ell ^6 \biggl [\left( 30 n^3+342 n^2+984 n+784\right) \sqrt{3 (\ell -1) (\ell +3) \tilde{Q}^2+16}+n^3 \left( 905-113 \tilde{Q}^2\right) \nonumber \\&\quad +n^2 \left( 5445-885 \tilde{Q}^2\right) +n \left( 10582-2050 \tilde{Q}^2\right) -1446 \tilde{Q}^2+6770\biggr ]\nonumber \\&\quad +\ell ^5 \biggl [4 \left( 45 n^3+335 n^2+784 n+576\right) \sqrt{3 (\ell -1) (\ell +3) \tilde{Q}^2+16}-2 n^3 \left( 59 \tilde{Q}^2-755\right) \nonumber \\&\quad +n^2 \left( 8716-1092 \tilde{Q}^2\right) -8 n \left( 427 \tilde{Q}^2-2228\right) -3057 \tilde{Q}^2+12135\biggr ]\nonumber \\&\quad +\ell ^4 \biggl [4 \left( 105 n^3+685 n^2+1437 n+987\right) \sqrt{3 (\ell -1) (\ell +3) \tilde{Q}^2+16}+n^3 \left( 1457-179 \tilde{Q}^2\right) \nonumber \\&\quad +n^2 \left( 9425-2127 \tilde{Q}^2\right) +n \left( 20518-6274 \tilde{Q}^2\right) -5385 \tilde{Q}^2+14535\biggr ]\nonumber \\&\quad +\ell ^3 \biggl [4 \left( 120 n^3+705 n^2+1366 n+899\right) \sqrt{3 (\ell -1) (\ell +3) \tilde{Q}^2+16}+n^3 \left( 988-676 \tilde{Q}^2\right) \nonumber \\&\quad +n^2 \left( 6710-4454 \tilde{Q}^2\right) +n \left( 14492-9372 \tilde{Q}^2\right) -6600 \tilde{Q}^2+10348\biggr ]\nonumber \\&\quad +\ell ^2 \biggl [2 \left( 59 n^3+295 n^2+588 n+468\right) \sqrt{3 (\ell -1) (\ell +3) \tilde{Q}^2+16}\nonumber \\&\quad -(n+2) \left( n^2 \left( 723 \tilde{Q}^2+97\right) +3 n \left( 723 \tilde{Q}^2+97\right) +1956 \tilde{Q}^2-504\right) \biggr ]\nonumber \\&\quad +\ell \biggl [2 (n+2) \left( n^2 \left( 9 \tilde{Q}^2-565\right) +3 n \left( 9 \tilde{Q}^2-565\right) -4 \left( 9 \tilde{Q}^2+266\right) \right) \nonumber \\&\quad -4 \left( 61 n^3+305 n^2+476 n+220\right) \sqrt{3 (\ell -1) (\ell +3) \tilde{Q}^2+16}\biggr ]\nonumber \\&\quad -120 (n+1) (n+2)^2 \sqrt{3 (\ell -1) (\ell +3) \tilde{Q}^2+16}+12 (n+1) (n+2)^2 \left( 9 \tilde{Q}^2-41\right) \biggr \}, \end{aligned}$$

The results for $\tilde{Q}=0$ are simple. For example, we have

C.3 $$\begin{aligned} \begin{aligned} \omega _3^+(\ell ,n,0)&=\,-\frac{n+1}{\ell ^2 (\ell +1)^5 (\ell +2)^5 \left( \ell ^4+4 \ell ^3-7 \ell ^2-22 \ell +24\right) }\biggl \{15 \ell ^{16} \left( 25 n^3+75 n^2+86 n+36\right) \\&\quad +\ell ^{15} \left( 756 n^4+9024 n^3+23666 n^2+25924 n+10635\right) \\&\quad +3 \ell ^{14} \left( 154 n^5+4550 n^4+30365 n^3+70985 n^2+74362 n+29891\right) \\&\quad +\ell ^{13} \left( 6468 n^5+101412 n^4+496578 n^3+1049874 n^2+1052916 n+414787\right) \\&\quad +\ell ^{12} \left( 35196 n^5+385956 n^4+1522114 n^3+2925382 n^2+2804644 n+1082869\right) \\&\quad +\ell ^{11} \left( 86016 n^5+693552 n^4+2153328 n^3+3600596 n^2+3216904 n+1210625\right) \\&\quad +\ell ^{10} \left( 39760 n^5-87904 n^4-1511886 n^3-3906094 n^2-4101644 n-1592257\right) \\&\quad +\ell ^9 \left( -289856 n^5-3115816 n^4-12385724 n^3-23675676 n^2-22375192 n-8462247\right) \\&\quad -3 \ell ^8 \left( 246260 n^5+2183260 n^4+7783923 n^3+14151321 n^2+13125202 n+4960511\right) \\&\quad -2 \ell ^7 \left( 363888 n^5+3001290 n^4+10332156 n^3+18697255 n^2+17577854 n+6802284\right) \\&\quad +\ell ^6 \left( -132894 n^5-1063362 n^4-4100041 n^3-9041805 n^2-10390274 n-4784172\right) \\&\quad +6 \ell ^5 \left( 65346 n^5+520542 n^4+1624863 n^3+2471951 n^2+1820470 n+509992\right) \\&\quad +8 \ell ^4 \left( 53286 n^5+426288 n^4+1365032 n^3+2189278 n^2+1759685 n+567058\right) \\&\quad +88 \ell ^3 \left( 2442 n^5+19536 n^4+62751 n^3+101301 n^2+82270 n+26912\right) \\&\quad +16 \ell ^2 \left( 3623 n^5+28984 n^4+92786 n^3+148729 n^2+119432 n+38428\right) \\&\quad +32 \ell (n+2)^2 \left( 241 n^3+964 n^2+1295 n+572\right) +384 (n+1)^2 (n+2)^3\biggr \}, \end{aligned} \end{aligned}$$

C.4 $$\begin{aligned} \omega _3^-(\ell ,n,0)&=\,-\frac{3 (n+1)}{\ell ^2 (\ell +1)^5 (\ell +2)^5 \left( \ell ^2+2 \ell -8\right) \left( \ell ^2+2 \ell -3\right) ^2}\biggl \{5 \ell ^{18} \left( 25 n^3+75 n^2+86 n+36\right) \nonumber \\&\quad +\ell ^{17} \bigl (252 n^4+3258 n^3+9092 n^2+10408 n+4425\bigr ) \nonumber \\&\quad +\ell ^{16} \bigl (154 n^5+5054 n^4+37966 n^3+99224 n^2+113304 n+48897\bigr ) \nonumber \\&\quad +\ell ^{15} \bigl (2464 n^5+45188 n^4+260752 n^3+643190 n^2+731724 n+320220\bigr ) \nonumber \\&\quad +\ell ^{14} \bigl (17262 n^5+236610 n^4+1168195 n^3+2740075 n^2+3099718 n+1373280\bigr ) \nonumber \\&\quad +\ell ^{13} \bigl (69188 n^5+793636 n^4+3535114 n^3+7927998 n^2+8890460 n+3976230\bigr ) \nonumber \\&\quad +2 \ell ^{12} \left( 84154 n^5+850862 n^4+3500778 n^3+7501860 n^2+8287284 n+3724179\right) \nonumber \\&\quad +4 \ell ^{11} \left( 53928 n^5+472806 n^4+1739794 n^3+3425097 n^2+3627146 n+1622127\right) \nonumber \\&\quad +\ell ^{10} \left( -66724 n^5-1278476 n^4-7123933 n^3-17955107 n^2-21145974 n-9529848\right) \nonumber \\&\quad +\ell ^9 \left( -985096 n^5-10656428 n^4-46400242 n^3-101836448 n^2-111953856 n-49214295\right) \nonumber \\&\quad +\ell ^8 \left( -2469102 n^5-25776570 n^4-108825394 n^3-230903248 n^2-245888248 n-105114255\right) \nonumber \\&\quad -2 \ell ^7 \left( 1879384 n^5+19630910 n^4+81628616 n^3+169096401 n^2+175208146 n+72767760\right) \nonumber \\&\quad +\ell ^6 \left( -3592058 n^5-37293334 n^4-153137219 n^3-313529871 n^2-321194878 n-131607588\right) \nonumber \\&\quad -2 \ell ^5 \left( 576222 n^5+6337866 n^4+28465433 n^3+64296769 n^2+71910306 n+31549320\right) \nonumber \\&\quad +24 \ell ^4 \left( 72936 n^5+583488 n^4+1719502 n^3+2252078 n^2+1174821 n+95490\right) \nonumber \\&\quad +72 \ell ^3 \left( 22354 n^5+178832 n^4+568471 n^3+897557 n^2+700422 n+212400\right) \nonumber \\&\quad -432 \ell ^2 \left( 1217 n^5+9736 n^4+28882 n^3+38531 n^2+21768 n+3636\right) \nonumber \\&\quad -23328 \ell (n+2)^2 \left( 41 n^3+164 n^2+199 n+76\right) -279936 (n+1)^2 (n+2)^3\biggr \}. \end{aligned}$$

C.5 $$\begin{aligned} \begin{aligned} c_2^+(\ell ,n)=&\,-\frac{n+1}{\ell ^3 (\ell +1)^3 (\ell +2)^3}\times \\&\bigl [10 \ell ^8 \left( 10 n^2+20 n+11\right) +\ell ^7 \left( 255 n^3+1565 n^2+2473 n+1243\right) \\&+\ell ^6 \left( 210 n^4+2625 n^3+9495 n^2+12711 n+5909\right) \\&+\ell ^5 \left( 1260 n^4+10200 n^3+29120 n^2+34849 n+15217\right) \\&+\ell ^4 \left( 2820 n^4+19230 n^3+48822 n^2+54519 n+22769\right) \\&+\ell ^3 \left( 2880 n^4+18495 n^3+44813 n^2+48454 n+19744\right) \\&+\ell ^2 \left( 1362 n^4+8853 n^3+21515 n^2+23206 n+9384\right) \\&+18 \ell \left( 18 n^4+117 n^3+283 n^2+302 n+120\right) \\&+12 (n+2)^2 \left( 2 n^2+5 n+3\right) \bigr ]. \end{aligned} \end{aligned}$$

C.6 $$\begin{aligned} c_2^-(\ell ,n)=&\,-\frac{n+1}{(\ell -1) \ell ^3 (\ell +1)^3 (\ell +2)^3 (\ell +3)}\times \nonumber \\&\bigl [10 \ell ^{10} \left( 10 n^2+20 n+11\right) +\ell ^9 \left( 255 n^3+1765 n^2+3017 n+1607\right) \nonumber \\&+\ell ^8 \left( 210 n^4+3135 n^3+13189 n^2+20081 n+10337\right) \nonumber \\&+\ell ^7 \left( 1680 n^4+16701 n^3+56375 n^2+79108 n+39514\right) \nonumber \\&+\ell ^6 \left( 6150 n^4+51627 n^3+157937 n^2+211444 n+103060\right) \nonumber \\&+\ell ^5 \left( 13380 n^4+105779 n^3+315065 n^2+411681 n+195551\right) \nonumber \\&+\ell ^4 \left( 18822 n^4+151993 n^3+448163 n^2+568637 n+260789\right) \nonumber \\&+\ell ^3 \left( 17448 n^4+134151 n^3+375413 n^2+455782 n+202128\right) \nonumber \\&+\ell ^2 \left( 2250 n^4+13553 n^3+39135 n^2+57494 n+31736\right) \nonumber \\&-2 \ell \left( 6318 n^4+40315 n^3+93509 n^2+92906 n+33208\right) \nonumber \\&-12 (n+2)^2 \left( 486 n^2+1151 n+665\right) \bigr ]. \end{aligned}$$

C.7 $$\begin{aligned} c_3^+(\ell ,n)&=-\frac{n+1}{(\ell -2) (\ell -1) \ell ^5 (\ell +1)^5 (\ell +2)^5 (\ell +3) (\ell +4)}\times \nonumber \\&\quad \bigl [15 \ell ^{17} \left( 91 n^3+273 n^2+303 n+121\right) +\ell ^{16} \left( 5040 n^4+43365 n^3+104335 n^2+106385 n+40583\right) \nonumber \\&\quad +\ell ^{15} \left( 7056 n^5+115920 n^4+571320 n^3+1160680 n^2+1092959 n+398063\right) \nonumber \\&\quad +\ell ^{14} \left( 3696 n^6+128016 n^5+1131144 n^4+4134936 n^3+7325376 n^2+6398001 n+2223169\right) \nonumber \\&\quad +\ell ^{13} \left( 51744 n^6+955136 n^5+6005776 n^4+17895274 n^3+28166734 n^2+22848697 n+7555941\right) \nonumber \\&\quad +\ell ^{12} \left( 281568 n^6+3649184 n^5+17991064 n^4+45140146 n^3+63047590 n^2+46982515 n+14607239\right) \nonumber \\&\quad +\ell ^{11} \left( 688128 n^6+6587840 n^5+24621856 n^4+47336904 n^3+51182552 n^2+29855341 n+7361149\right) \nonumber \\&\quad +\ell ^{10} \left( 318080 n^6-767424 n^5-19740056 n^4-79113504 n^3-140832704 n^2-121424797 n-41453349\right) \nonumber \\&\quad +\ell ^9 \left( -2318848 n^6-29467424 n^5-147334992 n^4-381111663 n^3-546478093 n^2\right. \nonumber \\  &\quad \left. -414870374 n-130499224\right) \nonumber \\&\quad +\ell ^8 \left( -5910240 n^6-62116320 n^5-273254136 n^4-647699319 n^3-874604437 n^2\right. \nonumber \\  &\quad \left. -636568664 n-194296946\right) \nonumber \\&\quad -8 \ell ^7 \left( 727776 n^6+7124306 n^5+29834722 n^4+68538983 n^3+90889405 n^2+65539020 n+19928374\right) \nonumber \\&\quad -8 \ell ^6 \left( 132894 n^6+1262738 n^5+5557318 n^4+14238422 n^3+21585941 n^2+17741480 n+6050763\right) \nonumber \\&\quad +8 \ell ^5 \left( 392076 n^6+3709140 n^5+14451756 n^4+29622189 n^3+33610805 n^2+19956976 n+4822764\right) \nonumber \\&\quad +8 \ell ^4 \left( 426288 n^6+4049736 n^5+15980758 n^4+33537407 n^3+39480989 n^2+24717280 n+6426852\right) \nonumber \\&\quad +64 \ell ^3 \left( 26862 n^6+255189 n^5+1007612 n^4+2117248 n^3+2497347 n^2+1567656 n+408996\right) \nonumber \\&\quad +32 \ell ^2 \left( 14492 n^6+137674 n^5+542450 n^4+1134745 n^3+1329113 n^2+826216 n+212820\right) \nonumber \\&\quad +128 \ell (n+2)^2 \left( 482 n^4+2651 n^3+5428 n^2+4894 n+1635\right) +1536 (n+1)^2 (n+2)^3 (2 n+3)\bigr ]. \end{aligned}$$

C.8 $$\begin{aligned} c_3^-(\ell ,n)&=-\frac{n+1}{(\ell -2) (\ell -1)^2 \ell ^5 (\ell +1)^5 (\ell +2)^5 (\ell +3)^2 (\ell +4)} \nonumber \\ {\vspace{5pt}}&\quad \times \bigl [15 \ell ^{19} \left( 91 n^3+273 n^2+303 n+121\right) +\ell ^{18} \left( 5040 n^4+46095 n^3+115757 n^2+121939 n+47605\right) \nonumber \\ {\vspace{5pt}}&\quad +3 \ell ^{17} \left( 2352 n^5+42000 n^4+225689 n^3+494859 n^2+495762 n+189152\right) \nonumber \\ {\vspace{5pt}}&\quad +\ell ^{16} \left( 3696 n^6+142128 n^5+1412520 n^4+5799009 n^3+11424699 n^2+10912308 n+4066786\right) \nonumber \\ {\vspace{5pt}}&\quad +2 \ell ^{15} \left( 29568 n^6+636312 n^5+4683960 n^4+16242717 n^3+29346615 n^2+26790223 n+9742869\right) \nonumber \\ {\vspace{5pt}}&\quad +2 \ell ^{14} \left( 207144 n^6+3328344 n^5+20341608 n^4+62716827 n^3+105442501 n^2+92147897 n+32636935\right) \nonumber \\ {\vspace{5pt}}&\quad +2 \ell ^{13} \left( 830256 n^6+11129152 n^5+59853152 n^4+168793783 n^3+266333757 n^2\right. \nonumber \\  &\quad \left. +222562652 n+76400934\right) \nonumber \\ {\vspace{5pt}}&\quad +\ell ^{12} \left( 4039392 n^6+47469536 n^5+230529664 n^4+599814122 n^3\right. \nonumber \\ {\vspace{5pt}}&\quad \left. +884578094 n^2+698202612 n+228547600\right) \nonumber \\ {\vspace{5pt}}&\quad +\ell ^{11} \left( 5177088 n^6+52118560 n^5+217844000 n^4+485424457 n^3\right. \nonumber \\ {\vspace{5pt}}&\quad \left. +601686747 n^2+388799865 n+100990311\right) \nonumber \\ {\vspace{5pt}}&\quad +\ell ^{10} \left( -1601376 n^6-37249760 n^5-261651952 n^4-881056901 n^3\right. \nonumber \\ {\vspace{5pt}}&\quad \left. -1585967679 n^2-1466587949 n-544639571\right) \nonumber \\&\quad +\ell ^9 \left( -23642304 n^6-298886000 n^5-1569335248 n^4-4392315665 n^3\right. \nonumber \\&\quad \left. -6886678995 n^2-5703560870 n-1936097508\right) \nonumber \\&\quad +\ell ^8 \left( -59258448 n^6-718739088 n^5-3644026392 n^4-9824708115 n^3\right. \nonumber \\&\quad \left. -14784268801 n^2-11726896832 n-3812738890\right) \nonumber \\&\quad -8 \ell ^7 \left( 11276304 n^6+136527410 n^5+682793314 n^4+1800593477 n^3\right. \nonumber \\&\quad \left. +2636970447 n^2+2029697780 n+639473946\right) \nonumber \\&\quad -8 \ell ^6 \left( 10776174 n^6+129579554 n^5+639764146 n^4+1663890578 n^3\right. \nonumber \\&\quad \left. +2403039845 n^2+1822775504 n+565248175\right) \nonumber \\&\quad -24 \ell ^5 \left( 1152444 n^6+14589612 n^5+77389468 n^4+217003577 n^3\right. \nonumber \\&\quad \left. +335392665 n^2+268903168 n+86980532\right) \nonumber \\&\quad +8 \ell ^4 \left( 5251392 n^6+49431600 n^5+183224790 n^4+338430639 n^3\right. \nonumber \\&\quad \left. +319923477 n^2+137337136 n+16396436\right) \nonumber \\&\quad +192 \ell ^3 \left( 201186 n^6+1885275 n^5+7338400 n^4+15170254 n^3+17523387 n^2+10683048 n+2672428\right) \nonumber \\&\quad -32 \ell ^2 \left( 394308 n^6+3740566 n^5+14087966 n^4+26757055 n^3+26694575 n^2+12989272 n+2311628\right) \nonumber \\&\quad -384 \ell (n+2)^2 \left( 59778 n^4+324011 n^3+631772 n^2+528130 n+160591\right) \nonumber \\&\quad -4608 (n+1)^2 (n+2)^3 (1458 n+2059)\bigr ]. \end{aligned}$$

## Singularity structure of several black-hole spacetimes

We include a table presenting the singularity structure of several linear perturbations of different spacetime geometries, together with the corresponding instanton counting description. The geometries are asymptotically anti-de Sitter, but the singularity structure is the same for the corresponding asymptotically de Sitter spacetimes, the difference being in the imposed boundary conditions (and therefore in the relevant connection formulae to consider).

**Table:**

SPACETIME GEOMETRY	TYPE OF PERTURBATION	SINGULARITY STRUCTURE	INSTANTON COUNTING
Schwarzschild-AdS_4_	Scalar field (generic mass)	5 regular	One instanton parameter is small, while the other remains finite in the $\mu \rightarrow 0$ limit
Schwarzschild-AdS_4_	Conformally coupled scalar field, electromagnetic perturbation, vector-type gravitational perturbation	4 regular (compared to the previous case, radial infinity is a removable singularity)	One small instanton parameter in the $R_h\rightarrow 0$ limit
Schwarzschild-AdS_4_	Scalar sector of gravitational perturbation	5 regular (but not radial infinity)	In the $R_h\rightarrow \infty$ limit, there is at least one instanton parameter with norm 1
Schwarzschild-AdS_5_	Scalar field (generic mass)	4 regular	One small instanton parameter in the $\mu \rightarrow 0$ limit
Schwarzschild-AdS_7_	Scalar field	5 regular	One instanton parameter is small, while the other remains finite in the $\mu \rightarrow 0$ limit. The difference with respect to the SAdS_4_ case is that, in the large-ℓ regime, the quantization condition only involves the expansion in the small instanton parameter. The *K*-th correction in the ω expansion in μ can be found by considering the 4*K*-th instanton expansion
Kerr–AdS_4_	Scalar field (generic mass)	5 regular	The rotation parameter *a* is bounded by a function of the black-hole mass. In the small-*a* regime, the instanton counting is as in the SAdS_4_ case
Kerr–AdS_4_	Conformally coupled scalar field and “spin-*s* perturbations” in terms of Newman–Penrose scalar quantities	4 regular (compared to the previous case, radial infinity is a removable singularity)	One small instanton parameter in the $R_h\rightarrow 0$ limit

**Table:**

SPACETIME GEOMETRY	TYPE OF PERTURBATION	SINGULARITY STRUCTURE	INSTANTON COUNTING
Kerr–AdS_5_	Scalar field (generic mass)	4 regular	The rotation parameter *a* is bounded by a function of the black-hole mass. In the small-*a* regime, the instanton counting is as in the SAdS_5_ case
Kerr–AdS_7_ with a single rotation parameter; small rotation parameter a∼0	Scalar field (generic mass)	5 regular	The same as for SAdS_7_
Kerr–AdS_7_ with a single rotation parameter; large rotation parameter	Scalar field (generic mass)	5 regular	The rotation parameter is not bounded; we take $a\sim \infty$. The instanton parameters behave as $1/a^2$ and $\mu /a^2$. The angular part is Heun, with the two-step connection procedure, but with integer parameters
Kerr–AdS_7_ with a single rotation parameter; black-brane limit	Scalar field (generic mass)	4 regular. One of the roots of the original $\Delta _r(r)$, in the variable $y=r^2$, goes to infinity, but infinity remains regular.	One small instanton parameter, $t\sim \hat{\mu }+O(\hat{\mu }^2)$. The angular part becomes hypergeometric, and the only contribution of the separation constant is the term proportional to $a^2$
Kerr–AdS_9_ with a single rotation parameter; black-brane limit	Scalar field (generic mass)	5 regular	The connection is the easy one with two nearby points, i.e. a hypergeometric-like connection, but the relevant instanton parameter is t∼1/2. The angular part is still hypergeometric, as above
Kerr–${\text {AdS}}_{2n+1}$ with a single rotation parameter; black-brane limit	Scalar field (generic mass)	n+1 regular: radial infinity, r=0, and n-1 of the *n* roots of $\Delta _r(r)$ in the variable $y=r^2$	There are n-2 instanton parameters

**Table:**

SPACETIME GEOMETRY	TYPE OF PERTURBATION	SINGULARITY STRUCTURE	INSTANTON COUNTING
Reissner–Nordström-AdS_4_	Scalar field (generic mass)	5 regular (radial infinity and 4 roots of $r^2 f(r)$ in *r*; r=0 is not a singular point)	The instanton counting is as in the SAdS_4_ case. The confluence to the extremal limit would occur on the side of the small instanton parameter, while the other one would remain finite
Reissner–Nordström-AdS_4_	Electromagnetic perturbation and vector-type gravitational perturbation	5 regular (r=0 and 4 roots of $r^2 f(r)$ in *r*; r=∞ is not a singular point)	The instanton counting is as in the SAdS_7_ case: one instanton parameter is small, while the other is finite, but in the opposite way with respect to the scalar perturbation
Reissner–Nordström-AdS_5_	Scalar field (generic mass)	4 regular (radial infinity and 3 roots of $r^4 f(r)$ in the variable $y=r^2$)	In the small-*Q* regime, the instanton counting is as in the SAdS_5_ case
Reissner–Nordström-AdS in D=2n+1 dimensions, n>2	Scalar field (generic mass)	2*n* regular (radial infinity and 2n-1 roots of $r^{4n-2} f(r)$ in the variable $y=r^2$, where the relevant expression is $Q^2 + y^{n-2}\!\left( y^n + y^{n+1} - y\mu \right)$)	There are 2n-3 instanton parameters
Reissner–Nordström-AdS in D=2n+1 dimensions, n>2; black-brane limit	Scalar field (generic mass)	It remains as above, since one still has $Q^2 + y^{n-2}\!\left( y^n + y^{n+1} - y\mu \right)$.	There are 2n-3 instanton parameters

**Table:**

SPACETIME GEOMETRY	TYPE OF PERTURBATION	SINGULARITY STRUCTURE	INSTANTON COUNTING
Reissner–Nordström-AdS in D=2n dimensions, n>2	Scalar field (generic mass)	4n-3 regular (radial infinity and 4n-4 roots of $r^{4n-4} f(r)$ in the variable *r*)	There are 4n-6 instanton parameters
C-metric	Scalar field (generic mass)	The radial equation has 4 regular singularities; the angular one has 5 regular singularities.	In the small-α limit, where α is the acceleration parameter, the radial equation has one small instanton parameter. If *Q* is also small, where *Q* is the black-hole charge, the angular equation has one small and one large instanton parameter; the relevant connection is the hypergeometric-like one between 0 and *t*, with *t* small

## Footnotes

1 For differential equations of order higher than two, one needs to use Toda field theory [20].

2 These coefficients can also be derived using the topological string approach, as done in the examples of *SU*(*N*) with $N_f = 0$ [16] or SU(2) with $N_f = 1$ [17]. However, for the cases of interest here, the method based on crossing symmetry [19] is more straightforward and easier to implement. Let us also mention that yet another way of computing these coefficients can be obtained by extending [12] to open paths.

3 We suppress the dependence of $F\left( {:z_2\over z_1}\frac{z_3}{z_2}\dots ,\frac{z_{n-2}}{z_{n-3}}\right)$ on $a_i$’s and $b_i$’s in our notation since the instanton parameters imply the order of $a_i$’s and $b_i$’s.

4 We remark that the notation $a_{0\theta }$ inside the semiclassical conformal blocks does not denote a shift in the momentum anymore, but it specifies the two choices of the intermediate channel labeled by θ.

5 Up to an overall factor coming from (2.27) which we will fix in concrete examples below.

6 There are 16 possible dictionaries between the masses of the (anti-)fundamental hypermultiplets and the semiclassical momenta, which can be found by considering all the possible sign flips.

7 To be more precise, the crossing symmetry applies to correlators as in (2.31). Since we assume the symmetry passes to the integrand and all the structure constants *C* are the same on the two sides of the identity, we can relate conformal blocks by the same Jacobian factor.

8 We remark that this phase factor can change according to the choices of the (complex) values of the parameters. Here, we fixed it by matching the phases on both sides for real parameters, as shown in Tables 1 and 2. We also show in (3.20) an example involving complex parameters.

9 We use this hypothesis to perform the numerical checks, but we expect the formulae to hold for complex *t* and *q* too.

10 We can also use (3.13) for the solution around *t*. The choice (3.30) is to explain the calculation of connection coefficients more intuitively.

11 For now, we ignore the phase factor. We will fix it when we write down the explicit form that we use.

12 We are setting $G_N=1$.

13 It does not seem to be related to BH thermodynamics, e.g. to the Hawking–Page point.

14 We remark that in principle other dictionaries can be found, since the differential equation only depends on the square of the *a*-parameters and there are symmetries w.r.t. some permutations of the $a_i$’s parameters.

15 For the thermal two-point functions $G_R(\omega , \ell )$ instead we need not only $b_2$ but the full NS function. Consequently, an efficient expansion in μ is not possible for $G_R(\omega , \ell )$, as the instanton counting parameter *t* in this case cannot be made parametrically small.

16 Note that these poles in ℓ are artifacts of our expansion and are resolved once the non-perturbative effects in ℓ are taken into account.

17 This work appeared approximately one year after the first version of the present manuscript.
